# Bronchiolitis recovery and the use of High Efficiency Particulate Air (HEPA) Filters (The BREATHE Study): study protocol for a multi-center, parallel, double-blind, randomized controlled clinical trial

**DOI:** 10.1186/s13063-024-08012-0

**Published:** 2024-03-20

**Authors:** Kelly Cowan, Erin O. Semmens, Jeannette Y. Lee, Ethan S. Walker, Paul G. Smith, Linda Fu, Rosalyn Singleton, Sara McClure Cox, Jennifer Faiella, Laurie Chassereau, Lora Lawrence, Jun Ying, Jaime Baldner, Maryam Garza, Robert Annett, Sheva K. Chervinskiy, Jessica Snowden

**Affiliations:** 1 Department of Pediatrics, Larner College of Medicine at the University of Vermont, 111 Colchester Ave, Smith 5, Burlington, VT 05403 USA; 2 School of Public and Community Health Sciences, University of Montana, 177 Skaggs, Missoula, MT 59812-2016 USA; 3 University of Arkansas for Medical Sciences, 4301 West Markham, #781, Little Rock, AR 72205 USA; 4 National Institutes of Health Environmental Influences On Child, Health Outcomes (ECHO) Program, 11601, Landsdown Street, Rockville, MD 20852 USA; 5 Alaska Native Tribal Health Consortium, AIP-CDC, 4055 Tudor Centre Drive, Anchorage, AK 99508 USA; 6 University of Vermont, Given C421, 89 Beaumont Ave, Burlington, VT 05405 USA; 7 IDeA States Pediatric Network Data Coordination and Operations Center, 13 Children’s Way, Slot 512-35, Little Rock, AR 72202 USA; 8 Department of Family Medicine, School of Medicine, University of Colorado Anschutz Medical Campus, Mail Stop F496, Academic Office One L15-3407, 12631 E 17th Avenue, Aurora, CO 80045 USA; 9 Department of Biomedical Informatics, University of Arkansas for Medical Sciences, 4301 W Markham Street, Little Rock, AR 72205 USA; 10 University of New Mexico Health Sciences Center, Albuquerque, NM 87106 USA; 11 Cook Children’s Department of Immunology, 1500 Cooper St, Fort Worth, TX 76104 USA

**Keywords:** Bronchiolitis, HEPA, Air filtration, Indoor air quality, Particulate matter, PM_2.5_

## Abstract

**Background:**

Acute viral bronchiolitis is the most common reason for hospitalization of infants in the USA. Infants hospitalized for bronchiolitis are at high risk for recurrent respiratory symptoms and wheeze in the subsequent year, and longer-term adverse respiratory outcomes such as persistent childhood asthma. There are no effective secondary prevention strategies. Multiple factors, including air pollutant exposure, contribute to risk of adverse respiratory outcomes in these infants. Improvement in indoor air quality following hospitalization for bronchiolitis may be a prevention opportunity to reduce symptom burden. Use of stand-alone high efficiency particulate air (HEPA) filtration units is a simple method to reduce particulate matter ≤ 2.5 µm in diameter (PM_2.5_), a common component of household air pollution that is strongly linked to health effects.

**Methods:**

BREATHE is a multi-center, parallel, double-blind, randomized controlled clinical trial. Two hundred twenty-eight children < 12 months of age hospitalized for the first time with bronchiolitis will participate. Children will be randomized 1:1 to receive a 24-week home intervention with filtration units containing HEPA and carbon filters (in the child’s sleep space and a common room) or to a control group with units that do not contain HEPA and carbon filters. The primary objective is to determine if use of HEPA filtration units reduces respiratory symptom burden for 24 weeks compared to use of control units. Secondary objectives are to assess the efficacy of the HEPA intervention relative to control on (1) number of unscheduled healthcare visits for respiratory complaints, (2) child quality of life, and (3) average PM_2.5_ levels in the home.

**Discussion:**

We propose to test the use of HEPA filtration to improve indoor air quality as a strategy to reduce post-bronchiolitis respiratory symptom burden in at-risk infants with severe bronchiolitis. If the intervention proves successful, this trial will support use of HEPA filtration for children with bronchiolitis to reduce respiratory symptom burden following hospitalization.

**Trial registration:**

NCT05615870. Registered on November 14, 2022.

**Supplementary Information:**

The online version contains supplementary material available at 10.1186/s13063-024-08012-0.

## Introduction and background

### Introduction

#### Study rationale

Acute viral bronchiolitis is the most common reason for hospitalization of infants less than 2 years of age in the USA, with ~ 130,000 admissions per year [1, 2]. The prevalence of bronchiolitis is between 18 and 32% in the first year of life and between 9 and 17% in the second year of life [3, 4]. Children hospitalized for bronchiolitis are at high risk for shorter-term (recurrent respiratory symptoms and wheeze in the subsequent year) and longer-term (persistent childhood asthma) adverse respiratory outcomes for which there are no effective secondary prevention strategies. The majority of hospitalizations for bronchiolitis (78–87%) occur in children < 1 year old [1], among whom bronchiolitis constitutes 18% of all hospitalizations. In these children < 1 year old, there may also be higher risk of recurrent wheeze and development of asthma relative to older children hospitalized with bronchiolitis.

The early life event of the first episode of severe (hospitalized) bronchiolitis may be a critical time point to implement prevention strategies to reduce respiratory symptom burden in this high-risk population. Multiple factors, including environment, contribute to risks of adverse outcomes. Indoor air pollution is a known modifiable environmental risk factor for respiratory conditions, and improvement of IAQ following hospitalization for bronchiolitis may be a prevention opportunity to improve health outcomes.

Numerous treatments have been evaluated to prevent symptoms and longer-term respiratory effects in infants hospitalized for bronchiolitis [5–13], but no effective strategies have been identified to date. Observational studies have repeatedly indicated that the environment, and air pollution in particular, is an important target for intervention with decades of research showing that air pollution adversely impacts respiratory health [14–18]. Infants are particularly susceptible to respiratory impacts of air pollution because their lungs are not fully developed; they have a high respiratory rate, and their intake of air relative to bodyweight is greater compared to adults [19]. In healthy infants, associations have been observed between exposure to higher air pollution and increased risk of respiratory symptoms following respiratory tract infections as well as respiratory infections that are longer in duration [18]. In its 2021 policy statement, “Ambient air pollution: health hazards to children,” the American Academy of Pediatrics highlights the role of air pollution in respiratory diseases, lung development, and asthma incidence and the importance of reducing these harmful exposures [19].

Fine particulate matter (particulate matter < 2.5 microns in aerodynamic diameter; PM2.5) is one of the air pollutants most strongly and consistently linked to health effects. Ambient sources include traffic, industry, and wildfires. Examples of indoor sources include outdoor PM2.5 that has infiltrated appliances, woodstoves, and pets. Infants, on average, spend approximately 90% of their time indoors [20, 21]. As a result, it is critical to maximize the quality of indoor air.

Portable air cleaners (PACs) effectively reduce PM2.5 concentrations in indoor air, with the vast majority of studies indicating reductions of at least 50% [22]. PACs are appealing as interventions because they are commercially available and can be universally implemented. PACs do not disrupt home infrastructure and do not require specialized expertise or medical prescription. HEPA is a type of filter in a PAC that is highly efficient in removing PM2.5. In interventional trials, use of HEPA filters has been associated with improvement in respiratory outcomes such as asthma in children and chronic obstructive pulmonary disease (COPD) in adults. Specifically, HEPA filtration resulted in improvements in pulmonary function and asthma control test scores and decreases in asthma-related healthcare visits and symptom scores [23]. In a recent study of HEPA efficacy in former smokers with COPD, those assigned to the active filter group, relative to placebo, had greater reduction in respiratory symptoms and a lower rate of moderate exacerbations and rescue medication use after 6 months [24].

In summary, air pollution is associated with respiratory symptoms and disease, particularly in sensitive populations, including infants. Air pollution is, therefore, a key intervention target. HEPA filters are efficacious in cleaning the air and improving multiple indicators of health. To date, however, no clinical trial has tested the efficacy of HEPA filtration units in increasing symptom-free days (SFDs) in infants hospitalized for bronchiolitis. Our study aims to address this important gap and improve the health of infants who have experienced this severe and common respiratory event.

Reduction in these symptoms may lead to decreased healthcare utilization and improve QOL for a large population. The current bronchiolitis care guidelines lack recommendations for post-hospitalization symptom reduction. If effective, HEPA filtration intervention can help fill this gap.

*Research question*: For children < 12 months of age hospitalized with bronchiolitis, will those who receive a HEPA filtration unit household intervention to reduce PM2.5 have decreased respiratory symptom burden over 24 weeks compared to those who receive a control HEPA unit?

### Background

#### There is a high burden of respiratory sequelae for children hospitalized with bronchiolitis

In addition to the recognized morbidity and mortality associated with the acute infection, children often experience subsequent, recurrent respiratory symptoms with a high burden of symptomatic days, especially children who are less than 12 months old. Furthermore, 30–40% of children who are hospitalized for bronchiolitis progress to have recurrent episodes of wheezing with or without lower airway infections [25–27]. There is also an increased risk of these children developing asthma compared to children without a history of bronchiolitis [27, 28], with 30–50% of these children developing asthma by 5 years of age [29]. Data suggest that the airways can be affected into adulthood, resulting in an increased incidence of asthma and chronic obstructive pulmonary disease in those with a history of infantile bronchiolitis [30]. Bronchiolitis and recurrent wheezing in this age group also impact QOL. Domains that are negatively impacted include overall health, discomfort, and physical abilities of the child, and parental stress [31, 32]. The frequency of other respiratory illnesses, respiratory symptoms, and the parental impacts of increased anxiety and associated medical costs is also increased in families with a child who has bronchiolitis and recurrent wheeze [33].

#### Bronchiolitis is a heterogeneous disease in both presentation and later childhood outcomes, but post-acute recurrent respiratory symptoms are a common element

The case definition of acute bronchiolitis is based on clinical criteria. The American Academy of Pediatrics’ definition of infectious bronchiolitis includes children under the age of 2 years with “a constellation of clinical symptoms and signs including a viral upper respiratory prodrome followed by increased respiratory effort and wheezing” [34]. There is recognized heterogeneity of the disease presentation and outcomes. For example, most infected children are not admitted to the hospital, and only a small percentage require intensive care [35, 36]. Also, time to recovery varies from several days to persistent symptoms past the duration of infection [13, 37, 38]. Several conditions other than infections can present with wheezing in this age group, so there is confusion in the literature as several terms have been used interchangeably, such as reactive airway disease or infantile asthma or wheezy bronchitis [39]. Interestingly, there appears to be a dose–response relationship between the severity of the infectious episode and risk of recurrent wheeze as infants with bronchiolitis who are hospitalized are at increased risk for recurrent wheeze and asthma compared to those not hospitalized [40]. The viral load (based on quantitative analysis of genomic material in secretions) trends with the severity of illness [41, 42]. These data support the notion that reducing insults to the respiratory tract might have a long-term impact on airway health. Even though cases of bronchiolitis are heterogeneous, cases of bronchiolitis requiring hospitalization of *any* severity are a risk factor for recurrent respiratory symptoms and asthma.

A number of infectious agents are associated with bronchiolitis. The most commonly identified pathogens are respiratory syncytial virus (RSV) and rhinovirus (RV), although there are several other infectious agents, including influenza, human metapneumovirus, adenovirus, and uncommonly, *Bordetella pertussis* that can cause bronchiolitis [43]. Both the long-term sequelae and presentations of bronchiolitis appear to vary among agents, although there is significant overlap, including increased risk of recurrent respiratory symptoms in the subsequent year and increased preschool asthma risk. RSV (the most common etiology in infants less than 1 year of age) tends to present with more severe illness, increased risk of respiratory failure, and longer hospitalization than RV but may be associated with a lower incidence of longer-term wheezing and asthma compared with RV [44, 45]. RV, in turn, tends to have a milder course than RSV but the subsequent development of asthma that persists later in childhood is more common [46, 47]. It is unclear if the long-term consequences of bronchiolitis (recurrent wheezing or asthma) occur because of a genetic predisposition or as a result of damage to the airways from the initial or repeated infections, and what role household environment may play in exacerbating these factors. Certainly, the pathogenesis of bronchiolitis, regardless of infectious agent, could lead to long-term airway damage since the virally induced process causes airway inflammation and plugging from cellular necrosis and mucous. For safety reasons, it is impractical to sample small airway specimens from infants who have fully recovered from the acute infection to study the structural or cellular mechanisms accounting for repeated episodes of wheezing or asthma. Evidence for long-term pathologic changes can be extrapolated from a rat model of RSV bronchiolitis where airway inflammation and cellular debris in the acute phase of infection precede a prolonged period of airway remodeling with airways scarring, smooth muscle hypertrophy, and mucosal thickening. In this animal model, there are also increased numbers of eosinophils. These changes would implicate both structural narrowing and cellular- or cytokine-mediated sensitization to foreign antigens as mechanisms of wheezing post-recovery [48].

#### Environmental exposures, including indoor air quality, influence respiratory health and are unstudied targets for prevention of recurrent respiratory symptoms after bronchiolitis

Because there are no effective treatments for viral bronchiolitis and long-term effects can be serious and/or burdensome, disease sequelae prevention is important. Interventions that reduce the risk of recurrent wheeze and other respiratory symptoms after the initial episode can immediately affect the burden of illness on the child, family, and healthcare system. Interventions that disrupt harmful interactions among the host, subsequent respiratory viruses, and the environment might also impact the risk and severity of wheezing illness in the very young, and the long-term risk of airway damage and asthma. One preventative measure to reduce respiratory symptoms widely supported in the literature is avoidance of air pollution. Predisposition to bronchiolitis appears to increase with exposure to environmental air pollution from either outdoor or indoor sources [49–51]. Numerous studies show a clear contribution of indoor air pollution to childhood lung disease, including bronchiolitis, pneumonia, and asthma [50–52]. Of the six air pollutants regulated by the U.S. Environment Protection Agency (EPA), particulate matter (PM) is most frequently identified in causing or worsening conditions such as COPD, asthma, cardiovascular events, and infections in adults, and low birth weight, asthma, and lower respiratory tract infection (LRTI) in children. Similarly, methods of lowering both short- and long-term exposure lessen the ill effects of PM [53–61]. PM decreases are associated with improved health outcomes in children with asthma [62]. PM has various components depending on the source, including elemental carbon, semi-volatile organic compounds, and heavy metals, all of which have oxidative potential [63, 64]. PM also can contain antigenic particles from animal dander, mites, cockroaches, and mold spores, among others, each of which can provoke airway sensitization [65]. PM is also a major component of tobacco smoke with a separate set of components, but still with major health impacts [66, 67]. The type of PM most frequently associated with health impacts is particulate matter < 2.5 microns in aerodynamic diameter (PM2.5), which travels deep into the lungs and into the circulatory system [66]. Sources of indoor PM2.5 include infiltration of ambient PM [63, 66], as well as biomass combustion (from indoor or outdoor sources). PM2.5 generated from biomass combustion has a high percentage of carbonaceous material, which also has pro-oxidant properties [64, 66]. An extensive body of research indicates that it is the small size of these particles that is most important to respiratory health [68]. Unless removed, PM2.5 can persist in the air for extended periods of time.

A reliable method for decreasing PM2.5 in residential environments is portable air-cleaning units containing HEPA or HEPA-type filters [55, 69–75]. Most studies show reductions of 50% or greater [22]. In addition, larger-sized airborne particulate matter, such as pollen and dust, is also effectively cleared by HEPA filters. HEPA filters have greater than 90% removal efficiency for airborne particles from multiple sources between 0.001 and 10 microns in diameter [76]. In addition to a HEPA filter, the proposed system for this study, the Winix 5500–2, also contains a carbon filter, which removes gaseous pollutants including nitrogen dioxide [77], a combustion-generated pollutant and respiratory irritant.

Filter efficacy in removing particles, especially PM2.5, from the air has been demonstrated convincingly, but what is also clear is that the unit needs to be turned on for it to work. Filtration units that are too noisy or consume too much electricity may be unsustainable long term. We have selected the Winix 5500–2 for this study because it is relatively quiet, energy efficient, and with demonstrated efficacy in lowering PM2.5 concentrations.

We will measure home levels of PM2.5 in this study because it is a main component of indoor air pollution with a clear relationship to respiratory symptoms. It is the most likely component of indoor air pollution to be related to respiratory symptoms. PM2.5 is expected to be present in all homes, which is not true for all other types of air pollution. With the development of low-cost and easily installed sensors, it is now feasible to continuously measure and remotely monitor PM2.5 in homes [78–80]. In addition to HEPA filters, the filtration units used for this study’s intervention will also be equipped with carbon filters, a common component of stand-alone commercially available HEPA filtration units. Carbon filters may reduce exposure to non-PM indoor air pollutants, including NO_2_, thereby potentially enhancing the air-cleaning benefit of filtration units [77, 81]. Therefore, users of HEPA units additionally equipped with carbon filters may experience respiratory benefit even if PM2.5 is ultimately not the main or only factor driving symptoms.

Although it is beyond the scope of this protocol, children in this study may be followed longer term to determine whether this intervention reduces asthma rates or improves asthma outcomes. It is plausible that reducing respiratory symptom burden in early life, improved air quality in early life, or both can decrease childhood asthma rates after bronchiolitis.

#### Summary

Bronchiolitis and respiratory sequelae can cause lasting health and cost consequences with no currently identified effective secondary prevention. Accordingly, secondary preventive measures might significantly reduce the incidence of recurrent respiratory symptoms and long-term pulmonary consequences such as asthma. Indoor air pollution, specifically PM, affects airway health and is associated with childhood respiratory diseases. Therefore, this is a reasonable prevention target. However, it is unknown whether an intervention to reduce indoor air pollution can effectively reduce symptoms and improve symptom-free days among infants with severe bronchiolitis. Because HEPA filters reliably decrease these components of household air pollution and are easy and cost-effective to use, we propose to study HEPA filtration to decrease respiratory symptom burden in infants hospitalized with bronchiolitis.

### Methods/design

SPIRIT reporting guidelines were used in this manuscript (Chan A-W, Tetzlaff JM, Gøtzsche PC, Altman DG, Mann H, Berlin J, Dickersin K, Hróbjartsson A, Schulz KF, Parulekar WR, Krleža-Jerić K, Laupacis A, Moher D. SPIRIT 2013 Explanation and Elaboration: Guidance for protocols of clinical trials. BMJ. 2013;346:e7586).

Please refer to the additional file for the SPIRIT Checklist.

### Risk/benefit assessment

#### Known potential risks

This study poses minimal risk to participants. Possible risks include the following:

• There is potential for false reassurance that the intervention prevents all adverse home environmental exposures.
• Noise produced by the device may be considered by some to be a “white noise” but could be bothersome or harmful if the highest setting is used continuously in close proximity to the child.
• The device may take up space, causing inconvenience.
• Participating individuals could be injured or experience electrical shock during instrument installation or use in the home (childproofing required).

#### Known potential benefits

There is potential for benefit to the research community and future patients. There is the potential benefit to individual participants in decreasing respiratory symptoms, though we cannot estimate the direct impact on the health of the individual participants. Interventions provided in the study can improve the health of individuals living within the home environment, which may be beneficial to household members beyond the child participant (though this endpoint is not studied). HEPA filtration decreases PM in the air to improve IAQ, but we cannot estimate the direct impact on the health of the individual participants and household members.

#### Assessment of potential risks and benefits

We do not anticipate significant health risks to participants and will minimize the possible risks described above. The benefit of understanding relationship between the IAQ environment and the health of children with bronchiolitis outweighs the risks. Standard of care medication/treatment will not be altered based on study measurements. To minimize potential risks to participants:

• The study team will provide education that HEPA filtration will not prevent all adverse environmental exposures. Even though HEPA filtration can improve IAQ, it does not decrease all of the harmful contaminants that can be in the indoor environment. The study team will also emphasize that it is not known if the intervention provides any clinical benefit.
• The HEPA unit chosen produces less noise and takes less space than some other available units. In the recommended “high” setting, the noise generated is below the American Academy of Pediatrics recommendation for sound level in a neonatal intensive care unit [82] and quieter than typical speech and rainfall [83]. We will instruct parent(s)/guardian(s) not to use the max setting and to set up the filtration unit at least 5 feet from where the child sleeps. In addition, these instructions will be placed on a label attached to the filtration unit.
• The study team will guide equipment setup, maintenance, and safe use.

### Objectives and endpoints

#### Primary objective

To determine if use of a HEPA filtration unit home intervention reduces the respiratory symptom burden (symptom-free days; SFD) for 24 weeks compared to a use of a control unit.

Hypothesis: Children who receive a HEPA filtration home intervention after their first hospitalization with bronchiolitis will have a greater mean number of SFDs over 24 weeks compared to controls.

Endpoint: Number of caregiver-reported SFDs over 24 weeks following the child’s first hospitalization for bronchiolitis (SFD defined as a 24-h period without coughing, wheezing, or trouble breathing).

Justification: Children hospitalized for bronchiolitis have a large burden of symptomatic days over the subsequent year after hospitalization, with the majority of the symptom burden occurring over the first 6 months. Clinically, it is important for the intervention to reduce the number of symptomatic days.

#### Secondary objective 1

To test the efficacy of HEPA filtration home intervention, relative to the control arm, on difference in number of unscheduled healthcare visits for respiratory symptoms over 24 weeks.

Hypothesis: Children who receive a HEPA filtration home intervention after their first hospitalization with bronchiolitis will have fewer unscheduled healthcare visits for respiratory symptoms over 24 weeks (lower number of hospitalizations, ED or UC visits, and other medical visits) compared to the control.

Endpoint: Caregiver-reported number of hospitalizations, ED or UC visits, or other unscheduled medical visits for respiratory complaints (cough, wheeze, or trouble breathing).

Justification: Children hospitalized for bronchiolitis are prone to recurrent respiratory symptoms. As a result, some children need hospitalization, emergency or urgent care visits, or other unscheduled medical visits for these symptoms. If the intervention can reduce healthcare visits (by reducing respiratory symptoms), this may lead to considerable cost savings.

#### Secondary objective 2

To test the efficacy of a HEPA filtration home intervention, relative to the control arm, on difference in QOL.

Hypothesis: Child QOL will be higher in families that receive the HEPA intervention compared to controls.

Endpoint: Total QOL score, as measured by the PedsQLTM Pediatric Quality of Life Inventory Infants Scales.

Justification: Children hospitalized for bronchiolitis can have decreased QOL post-hospitalization for months or longer due to a variety of factors, including ongoing or recurrent respiratory symptoms, or impact on the family of the experience of the child’s severe illness requiring hospitalization. Increased child QOL is expected to follow an intervention that improves respiratory symptoms.

#### Secondary objective 3

To test the efficacy of HEPA filtration home intervention, relative to the control arm, on difference in PM2.5 levels in the home over 24 weeks.

Hypothesis: PM2.5 levels will be lower in households that receive the HEPA intervention compared to controls.

Endpoint: Average PM2.5 levels as measured by 2 in-home PurpleAir monitors during the 24-week intervention period and scaled to the unit of μg/m^3^ per week.

Justification: To demonstrate that a putative agent causing increased susceptibility to recurrent wheeze in infants is being reduced by active HEPA filtration.

PM2.5 is one of the most heavily studied criteria pollutants for causing lung disease.

### Study design

#### Overall design

This is a multi-center, parallel, double-blind, randomized controlled clinical trial. Two hundred eighteen children < 12 months old with their first hospitalization for bronchiolitis will be randomized 1:1 (stratified by site) to receive 24 weeks of home intervention with active HEPA filtration units to improve IAQ or to a control group without a HEPA or carbon filter inside identical-appearing units. Children will be followed for respiratory symptoms during a pre-intervention period of up to 2 weeks following randomization and during an intervention period of 24 weeks.

This study is designed to reduce barriers to participation for rural participants in that there will be no required study visits to a distant study site, and all study activities and data collection will be conducted remotely. Participants will be identified in hospitals in ISPCTN states, maximizing the chances that rural and medically underserved populations are represented. It is common for rural children with bronchiolitis to be transferred to tertiary care centers in urban/suburban locales, so inclusion of urban hospitals will allow for recruitment of this population [1]. It is important for rural children to be represented in a bronchiolitis study in order to increase generalizability. Rural and underserved children have a higher risk of decreased access to medical care for symptoms and illness episodes, and a higher burden of asthma [84, 85]. These families may have air pollutant exposure profiles distinct from those residing in urban areas. For example, they might experience less exposure to traffic-related pollutants but may have more wood stove use or exposure to agricultural pollutants or wildfires. With its diversity of sites, the ECHO ISPCTN is well-positioned to enroll rural children that might otherwise be excluded.

#### Scientific rationale for study design

We propose a parallel, randomized controlled trial (RCT) as the most scientifically robust design to determine the efficacy of HEPA filtration in improving the number of SFD over 24 weeks following hospitalization for bronchiolitis. We considered two alternative study designs to increase acceptability of an inactive filtration unit: randomized crossover and stepped wedge. Parallel, crossover, and stepped wedge designs each allow for a control or placebo group, which is critical as there is genuine uncertainty regarding the efficacy of the intervention in reducing SFDs in the 6 months following hospitalization for bronchiolitis. The parallel design is distinct from the other two in that homes randomized to the control arm will not receive the intervention during follow-up.

Both the crossover and stepped wedge designs are appealing in that they allow all participants to receive an intervention that we expect will improve IAQ. However, with a crossover design, it is critical that the participant’s disease characteristics are the same at time zero of each time period. Since the eligibility for this study is based on hospitalization for bronchiolitis, there is no way that can be achieved. Moreover, the relevant time window of exposure, washout duration, and appropriate point in time to crossover is unclear. Although an early benefit of HEPA filtration is possible, the intervention may be more effective over a longer duration rather than the immediate period post-hospitalization, which would require a lengthy study.

The stepped wedge is a variant of an interrupted time series design in that a site starts in the control arm and switches over to the intervention arm at a specific point in time. While participants may be blinded, staff are not, so this might be difficult to implement.

We considered a 1-year study intervention period. However, due to family burden, risk of missing data, and risk of nonadherence to the intervention over such a long duration, we ultimately decided that a 24-week intervention targeting the time period of highest respiratory burden was preferable. Although a 1-year study period is appealing due to capturing potential variability in air quality (heating season, etc.) and viral exposures (cold seasons), the severity of respiratory symptoms is not static in rapidly growing infants, and most of these children will be recruited in the same seasons and have similar opportunity for repeat viral exposures and heating seasons between the groups.

We considered including children up to 2 years of age (per the definition of bronchiolitis). However, the under 12 months age group has the highest symptom burden and likelihood of demonstrating an effect.

We chose the primary outcome of symptom-free days because assessing the number of wheezing episodes alone can underestimate the burden of chronic symptoms (including cough) and prolonged symptoms with illness episodes. In addition, clinically it can be difficult to determine the discrete number of wheezing episodes for children with prolonged or chronic wheeze (which is a higher risk in this population).

The KidsAir study at the ISPCTN Montana site successfully implemented and completed a study similar in design to the one we propose here [86]. The proposed study benefits from methods used in the KidsAir study, lessons learned, and the study team’s expertise. Although the current study population is different from that of the KidsAIR study, the KidsAIR study targeted for intervention the same exposure to PM2.5 as the current study using the same method, namely HEPA filtration, and collected similar covariate data successfully over two winters of study participation [87]. In the KidsAIR study, field technicians visited homes approximately six times per winter season. Data collection procedures were more burdensome for participants and more frequent than those proposed here; however, participant retention in the other study was still 87% in the first year of the 2-year KidsAIR study. The current study requires a relatively shorter duration of study participation (6 months versus 2 years) and less burdensome procedures for outcome ascertainment that do not require participating families to accommodate home visits by field technicians.

The HEPA unit intervention will take place over the approximately 6 months after hospitalization because this period is when the majority of post-bronchiolitis respiratory symptoms occur [5, 26, 31]. The intervention involves the placement of two HEPA units within the home. The rationale for the placement of one HEPA unit in the child’s sleep space is that infants typically spend a continuous number of hours daily in this space. The rationale for placement of a second HEPA unit in a common area of the home is to increase the child’s exposure to the intervention during waking hours.

Schedule of activities (SPIRIT Figure).

**Table:** 

Evaluation/procedures	Screen_1_ (hospital)	Enroll / randomize In hospital (+ 1 week)	Pre-intervention Weeks 1–2 after hospital discharge_2_	Intervention_2_ Weeks 3–26	Intervention Week 26
Review inclusion/exclusion criteria	x	x			
Informed consent		x			
Document participant characteristics and risk factors for recurrent wheeze		x			x
Pre-intervention period (in all study participant homes—both intervention and control): up to 2 weeks continuous home PM_2.5_ monitoring via PurpleAir_3_			x		
Intervention period (in all study participant homes—both HEPA/control unit): Continuous home PM_2.5_ monitoring via PurpleAir_3_				x	
Continuous HEPA/control unit use_4_				x	
Continuous use of kilowatt meter to measure HEPA/control unit adherence_4_				x	
Weekly submission: Symptom survey, number of medical visits, number of nights away from home, HEPA/control unit adherence_5_			x	x	
Check-in contact with study team_6_			x	x	
QOL Survey_5_					x

^1^Screening and enrollment ideally will occur during hospitalization. However, enrollment can occur after discharge to home *if the family can receive and set-up the air quality monitoring equipment ideally within 7 days of discharge.* Other procedures can occur at home

^2^Day of hospital discharge is defined as day 1. Intervention ideally starts on day 14

^3^Families place PurpleAir monitors in the child’s sleep space and in another common room. Baseline PM2.5 measurements are collected for up to 14 days and then the family will begin using HEPA units in the same rooms (child’s sleep space and another common room) that contain the PurpleAir monitors while PM2.5 monitoring continues. HEPA units will have active filters in the intervention group and no HEPA or carbon filters in the control group

^4^Kilowatt hour meter is used to measure actual HEPA unit use. All devices are simple to plug in. The study team will work with the family remotely to confirm correct installation and placement of the devices at baseline and at the start of HEPA use and confirm data transmission from the PurpleAir monitor

^5^Family will receive an Electronic Data Capture (EDC) system survey link weekly by text (if allowed by the local site) or email. The family will submit the brief questionnaire (alternatively, the study staff can call the parent(s)/guardian(s) to read the questions and record the responses in the EDC system for the parent(s)/guardian(s)). QOL surveys will also be administered electronically (with alternative of survey completion by phone with study staff)

^6^ Weeks 1–4, check-in with the enrolling site will occur weekly and as needed (minimum of weekly). Weeks 5–26, check-in with the enrolling site will occur weekly or monthly and as needed (minimum of monthly). During the check-in, the study team will assist or prompt EDC documentation as needed, assess equipment questions/concerns, and safety assessments will occur (AE, SAE, UPIRTSO)

#### End of study definition

An individual participant will be considered to have completed the study after completing all final protocol-specified assessments at the end of the 24-week intervention period (which is equal to week 26 in SOA due to approx. 2 week pre-intervention period). Participants will be considered not to have completed the study if consent was withdrawn or the subject was lost to follow-up without submitting all end-of-study questionnaires (at end of 24 weeks of intervention) and surveys. The end of study (“study completion”) is defined as the date the last protocol-specified visit/assessment (including telephone contact and receipt of questionnaires and surveys) is completed for the last participant in the study. Scheduled study activities are shown in the Schedule of Activities (SOA).

### Study population

#### Inclusion criteria

To be eligible to participate in this study, an individual child must meet all of the following criteria:

- Age < 12 months at hospital admission
- First-time hospitalization for bronchiolitis
- One primary residence (> 5 days per week)
- Parent, legal guardian or other legally authorized representative consents to allow their child to participate and agrees to participate in all study activities
- Electricity in the home (required to power the study equipment)
- Wireless internet access or cellular service access in the home
- English- or Spanish-speaking parent or guardian

#### Exclusion criteria

An individual child who meets any of the following criteria will be excluded from participation in this study:

- Chronic airway or respiratory conditions requiring home oxygen, mechanical ventilation, or tracheostomy dependence; known immunodeficiency, hemodynamically significant cardiac conditions including those requiring medication or oxygen; cystic fibrosis; neuromuscular disease; eligible for palivizumab (per AAP guidelines) [88]
- Use of stand-alone home HEPA filtration other than study-related HEPA units in the home
- Household member who smokes (any type), vapes, or uses e-cigarettes
- Intention to move in the next 6 months
- Enrolled or plans to enroll in an interventional clinical trial for treatment of acute bronchiolitis or sequelae of bronchiolitis, unless permission is given by the PI
- Another child in the household is enrolled in this study (one child per household can enroll)

We will exclude homes with smokers to maximize our ability to determine the efficacy of the HEPA intervention in increasing SFDs. Multiple studies have demonstrated efficacy of HEPA filtration units in reducing non-nicotine particle-bound components of tobacco smoke [67, 73, 89–91]. However, we propose to exclude households with a smoker because even if HEPA filtration units reduce tobacco smoke components in the home, the secondhand smoke (SHS) exposures the child experiences with the smoker outside of the home (e.g., in the car) may be sufficient to reduce or eliminate any health benefits of the indoor HEPA unit. A systematic review and meta-analysis of 60 studies showed that passive smoke exposure is a major risk factor for lower respiratory tract infections and, in particular, bronchiolitis [92], as well as increased respiratory symptoms [93]. In addition, infants who live with a smoker may be exposed to more sources of SHS [94]. The exclusion of homes with smokers is consistent with the majority of RCTs that have evaluated the impact of portable air cleaners on health [22].

We acknowledge that excluding children living in households with a smoker will reduce the number of eligible participants. Nonetheless, including children from households with a smoker may increase sample size requirements if the intervention is less efficacious in smoking households [73, 95]. We emphasize that the proposed trial is an efficacy study. Our primary objective is to determine if HEPA filtration increases SFDs in children hospitalized for bronchiolitis under ideal circumstances. There is genuine uncertainty regarding this research question. Although smoking may be more prevalent in IDeA states, the percentage of homes with children and non-smokers is still clearly the majority, making the study still generalizable to a very large population of children with bronchiolitis.

If the intervention is efficacious, a next step would be to evaluate if these findings are generalizable to other populations, including children living in households with a smoker.

No children receiving concomitant medical therapies will be excluded.

#### Screen failures

A screen failure is a participant who, upon initial evaluation potentially meets inclusion criteria (e.g., through chart review) and does not appear to have any exclusion criteria, but who, upon further evaluation (e.g., discussion with parent(s)/guardian(s) about whether there is electricity in the house, whether anyone in the house smokes, whether they plan on moving within 6 months) prior to enrollment/randomization, does not meet either all of the inclusion criteria and/or has 1 or more exclusion criteria. Screen failure information will be collected and recorded on the appropriate case report form (CRF) and will include all reasons for the failure.

Individuals who do not meet the criteria for participation in this trial (screen failure) because of a modifiable factor may be rescreened. Rescreened participants should be assigned the same participant number as for the initial screening.

#### Strategies for recruitment and retention

Recruitment and retention of eligible participants will be critical to study success. Within a multi-center network, optimal recruitment approaches may vary from site to site, and network success may require sharing best practices among clinical sites. Though each clinical site is responsible for recruiting, enrolling, and retaining study participants, the Data Coordinating and Operations Center (DCOC) will assist each site in creating a site-specific recruitment and retention plan. Site initiation visits will include a review of the individual site’s recruitment and retention plans. All recruitment and retention materials, general and site-specific, must be approved by the IRB of record, which for all sites except Native American sites will be the UAMS IRB.

##### Screening/recruitment

Eligible children will be identified during a primary hospitalization for bronchiolitis. We will request a partial waiver of consent/HIPAA for the recruitment screening portion of the study, i.e., to allow sites to review medical records for potentially eligible participants that meet minimum inclusion criteria. This partial waiver will be required to reach the appropriate study population.

- *Screening*. Local sites will obtain a daily list or receive notification of admissions to their pediatric units with a diagnosis of bronchiolitis (of the days where the research team is available). This information will be obtained in accordance with individual institutional policies and procedures as well as IRB approval from the IRB of record. For non-Native American populations, the UAMS IRB (as central IRB), will be the IRB of record.
- *Recruitment*. Each potential participant on the list should be approached for recruitment if the child meets eligibility criteria from prescreening their medical record. Recruitment can occur in person or remotely in accordance with institutional requirements, family preference, and healthcare team approval.

Screening and enrollment ideally will occur during hospitalization, but enrollment can occur after discharge to home *if the family can receive and set up the air quality monitoring equipment within 7 days of discharge.* Other procedures can occur at home. Day of hospital discharge is defined as day 1.

If an eligible child is readmitted within 1 week of discharge to home from the initial hospitalization, this can be considered part of the same hospitalization and timeframe to obtain the consent (if not already done). The start of study activities resets to begin after the second discharge to home.

If a potential participant appears to meet eligibility requirements (i.e., based on pre-screening assessment) but declines participation, the parent(s)/legal guardian(s) will be asked why they do not want to participate. These data will be recorded in a screening log without any identifiers that could link responses back to an individual or family. The individual/family will be told they do not have to answer any questions they do not want to answer.

##### Action if recruitment is low/risk mitigation plan

Methods for screening and recruitment at individual sites will be reviewed to determine if there are gaps in offering the study or other factors leading to low recruitment. Sites will submit recruitment data (number of bronchiolitis admissions, number approached, number consented, reason for declining the study) to the DCOC every 2 weeks. Individual site action plans may be made to improve recruitment.

Using data from nine ISPCTN sites, we observed 3209 admissions for bronchiolitis in infants less than 12 months of age during the 2019–2020 season. We estimate that we will have a minimum of 10–14 sites in this trial. Therefore, we will have a population of approximately 3500–5000 infants hospitalized from bronchiolitis to recruit from per year. Given that the recruitment period for this study is estimated at 2 years, we should have 7,000–10,000 eligible infants during the study period in our recruitment sites. Assuming a conservative recruitment rate of 20% would give us 700–1000 infants, which is far greater than the recruitment targets for this trial. Additional sites could be added to the trial if recruitment falls short, as the ISPCTN has 18 awardees, some with multiple available recruitment sites.

##### Retention/incentives

Some studies reported decreased HEPA filtration unit usage over the course of follow-up [71, 96]. Study participants indicated noise and electricity costs as reasons for turning off the filtration unit or using it below the recommended setting. To address these challenges, we selected a filtration unit model that emits low decibels and has low electricity demands. A similar unit was used in the KidsAIR study. Evaluation of kilowatt (kW) meter data in KidsAIR indicated strong compliance with filtration unit usage recommendations. In addition, we will compensate participating families for electricity costs. Our intervention period of 6 months is favorable for adherence relative to a year(s)-long intervention. Additionally, coordinator contacts will provide problem-solving strategies and support for continued use of the HEPA filtration unit.

##### Coordinator contact for engagement while collecting study data

The research coordinator or other qualified research team member will check in with the family periodically. Check-in will typically be via phone call, but if permitted locally contact may occur by other means (such as text, email, video conferencing).

##### Weeks 1–4: check-in: contact from the enrolling site

- Weekly and as needed check-in: (site to conduct a weekly check-in at a minimum)
- Assist or prompt EDC documentation as needed
- Assess equipment set-up, questions/concerns
- Safety assessments (AE, SAE, UPIRTSO)

##### Weeks 5–26: check-in: contact from the enrolling site

- Monthly and as needed: (site and participant dependent)
- Assist or prompt EDC documentation as needed
- Assess equipment questions/concerns
- Safety assessments (AE, SAE, UPIRTSO)

##### Compensation will be provided for completion of study activities

- Weekly survey collection (26 submissions)—$20 per survey submitted (max $520 per participant)
- $10 for submitting QOL survey
- $5 each for baseline and 6-month (approx. week 26 of participation; week 24 of intervention) history question set (max $10)
- $40 for return of the PurpleAir monitors with internal SD cards, hotspot, and kW meter. This is for time spent returning equipment. Parent(s)/guardians of participant will receive pre-paid materials for returning equipment, i.e., at no expense to parent/guardian.

##### Compensation will be provided for anticipated excess energy costs

- $15 per family (The anticipated excess energy cost is approximately $5 per HEPA unit in higher energy cost areas. The $15 compensation will account for unanticipated energy costs.)
  - *Healthy Homes Kit*: All participants will receive a Healthy Homes Kit (approximately $42.00 value) near the start of their child’s study intervention period. The kit contains a collection of items to improve non-IAQ home environmental health and safety. The Healthy Homes Kit is a response to community and stakeholder feedback requesting meaningful home environmental tools in all study arms to make the study more acceptable. We do not expect an impact on the measured outcomes. The rationale for using the Healthy Homes Kit is that the home environment is generally considered important for overall health. The Kit addresses the following concerns: (1) recruitment and retention may be affected with a “placebo only” arm, and (2) when introduced to the rationale that a healthy home environment helps improve health, some families may want to pursue home environment modifications, and the kit will provide standard tools. The kit will contain children’s books, outlet covers, doorknob covers, cabinet and drawer latches, bath thermometer, carbon monoxide detector, bedbug traps, and green cleaning supplies.
  - *Additional incentive at study completion and equipment retained by families*: All families will retain the two HEPA units (value of $500) and receive a supply of two HEPA and carbon filters (value of $160). They also will keep the tape measure (value of $15), four surge protector power cords (value of $10 each) and one USB/AC power adapter (value of $4). They will keep their backpack (valued at approx. $22).

##### Compensation summary

- Total possible compensation (reimbursement for time and equipment return) for study activities: $580
- Compensation for excess energy costs: $15.
- Value (approx.) of equipment and supplies that families keep: $806.

Grand sum value of compensation is, therefore, approximately $1378.

Return of results: Indoor air quality results will not be provided to caregivers until after their child’s participation has ended (i.e., after 6 months, or sooner if the child’s participation ends early). After a participant completes the study, study staff will then send the participant’s caregiver a summary of the data from PurpleAir monitors in their home. Once the entire study is completed, study staff will provide a summary of the overall study results to caregivers of participants.

### Study intervention

#### Study interventions administration

##### Study intervention description

The 24-week intervention period captures the period of highest respiratory burden post-bronchiolitis.

Use of the HEPA units (experimental) or inactive (control) units takes place for 24 weeks beginning after a pre-intervention period approximately 2 weeks in duration (methods to be described in the MOP).

Since previous related work using a pre-/post-design has shown substantial variability in PM2.5 concentrations even in relatively small geographic areas, we will collect baseline PM2.5 to control for this potentially important source of variability in homes. For example, in a study in homes with wood stoves in the western U.S., baseline median PM2.5 was 17, 41, and 16 μg/m^3^ in filter, wood stove change out, and placebo arms, respectively [70]. Preliminary results from an ongoing RCT (NCT02240069) have shown a similar pattern with median baseline PM2.5 ranging from 23 to 41 to 30 μg/m^3^ in homes assigned to different intervention arms [97]. Note we will not exclude homes based on baseline PM2.5 measurements because even at low levels (i.e., below U.S. National Ambient Air Quality Standards), PM2.5 has demonstrated adverse health effects [98].

The rationale for placing the HEPA unit in the child’s sleep space is that the child will typically spend a continuous number of hours daily in this space. The rationale for a second HEPA unit in a common area of the home is to maximize exposure to the intervention.

Intervention for both experimental and control conditions:

- Use of the HEPA units (experimental) or control units takes place from weeks 3–26 (approximately) after hospital discharge to home.
- A coordinator or other qualified research team member will contact the family via video or phone to prompt them to begin using the HEPA or control units. The research team member will confirm proper installation and that the two units are functioning and provide information on the lights (e.g., sensor light) on the units.
- Both the HEPA and carbon filters will be removed from the control units, and interior contents of the unit will be masked with black cardstock or similar. The door on the unit will be taped closed to make it difficult to open the units.

##### Experimental condition, active HEPA filtration unit use

The intervention group will use two Winix 5500–2 HEPA filtration units. One will be placed in the child’s sleep space and one will be placed in another common room with both units running continuously on the “high” (i.e., level 3 / second from highest) setting. Each unit is 8.2 × 15.0 × 23.6 inches, and verified for a 360 sq. foot room. If a home is too small to accommodate 2 Winix units (for example, a single room residence), one Winix unit may be used for the study. Additional features beyond HEPA and carbon filter include plasmawave technology to reduce volatile organic compounds and odors. The plasmawave feature will be turned off to avoid ozone production.

##### Control condition, inactive filter unit use

The control group will use identical-appearing Winix 5500–2 units and identical setup procedures as described above, but with no HEPA or carbon filters.

The manufacturer indicates that HEPA filters can last up to 12 months. For this reason, the filters will not be changed during the 24-week intervention period. Changeout of filters also adds the additional risk of unintentional unmasking of the family or research team members. Ideally, the carbon filter in the HEPA unit is cleaned every 3 months. However, since reduction of pollutants (e.g., volatile organic compounds) addressed by the carbon filter are not targets of the intervention, the carbon filters will not be changed out during the 24-week study.

If a HEPA unit or control unit breaks, malfunctions, or stops working, the entire unit needs to be replaced by the study team as soon as possible (by mail, pickup, or delivery). While awaiting replacement, the family will be instructed to use the working HEPA unit in the room where the infant spends the most hours.

#### Preparation/handling/storage/accountability

##### Acquisition and accountability

All PurpleAir and related equipment to be used by participants in the experimental and control conditions will be stored at individual sites. Study equipment will be dispensed to participants prior to their discharge to home from their hospitalization (ideally), or if not possible by mail, or local pickup/delivery. Experimental and control filtration units and related equipment will be stored at the central site and, in most cases, be mailed directly to participants. Local sites may store filtration units if preferred and deliver them directly or by mail to participating families. Arrival of the equipment in the participants’ home will be confirmed by communication (including phone, text, or email) with the family by study staff, who will also arrange a time to assist with equipment set-up.

##### Formulation, appearance, packaging, and labeling

The HEPA and control units are identical in appearance. They will both have a standard manufacturer appearance externally with the modifications already described.

##### Product storage and stability

The HEPA/control units are prepared and stored centrally prior to dispensing to participants. PurpleAir monitors with hotspots are prepared centrally then stored by individual sites prior to dispensing to participants. Healthy Homes Toolkits will be prepared by the DCOC and then shipped in bulk to the central site prior to dispensing to participants.

##### Preparation

Education of parent(s)/guardian(s) of participants regarding equipment use will occur during the child’s hospitalization and then remotely (video, phone, or other family-preferred communication means) post-discharge to home. All participants will receive:

- 2 HEPA or control units (unless home is small enough that only one HEPA unit is needed),
- 2 kW meters (0 kW meters if home has only two-prong outlets),
- 2 PurpleAir monitors (1 if home using only one HEPA unit) and mobile hotspot with power adapter,
- 4 power strips or similar,
- 1 tape measure,
- Basic education on strategies to improve indoor air quality, and
- 1 Healthy Homes Kit.

The local site research team will assist participants remotely with setup of all study equipment.

Study staff will ensure correct placement and setup of the PurpleAir monitors and hotspot, HEPA/control units, and kW meter following a checklist.

Because parent(s)/guardian(s) of participants can set up all study equipment with remote support, the study team should not need to enter any participant’s home. However, to reduce barriers to participation, study staff will be permitted to assist any parent(s)/guardian(s) of participants in their home if all remote options for technical assistance are exhausted, and it is necessary to troubleshoot problems in person.

A checklist will be used to ensure all study equipment necessary for participation is dispensed to each participant’s family and that the requirements for correct equipment setup in the home are met. The checklist will be included in the study manual of procedures (MOP).

To facilitate the inclusion of rural children, this protocol allows for providing study equipment at hospital discharge to home, mailing equipment, or locally arranged pickup/delivery, and remote study activities. There are no required in-person study visits.

##### Measures to minimize bias

Randomization scheme:

Participants (within each site) will be randomized 1:1 (stratified by site) to receive HEPA filtration (intervention group) or control filtration. Permuted block randomization of participants (selected at random) will be employed. The block size and block permutation will be selected at random for each site. After selecting the block size and block permutation, a participant is assigned to the first control/intervention in the block, and the remaining slots are assigned as participants continue to randomize within the site. As randomizations continue and no more slots are available in the previously assigned block, a new block is assigned and participants are randomized accordingly. The mechanism of the allocation is computer-generated assignment. This assignment is not made until the eligibility of the participant and informed consent has been obtained. Both the participant and study staff at the site are masked as to which intervention was assigned. The study statistician generates the allocation sequence in advance of the study initiation and loads it into the electronic data capture system by the data coordinating center for use in the allocation. Site study staff enroll the participants and request the intervention assignment from the computer file.

##### Masking

Families will be masked as to whether their Winix units are equipped with or without HEPA/carbon filters.

Study coordinators, investigators, and other team members who interact with participants’ parent(s)/guardian(s) to obtain surveys, troubleshoot equipment setup and operation, or have other interactions will remain masked through the duration of the study for individual participants. This includes masking as to which intervention the participants receive and household air quality measurements, including the baseline measurements (separate personnel will need to be on the receiving end for air quality measurement data). This will require more than one study coordinator or additional staff/technician on the study team.

Unmasked personnel (separate coordinator, technician, or other qualified personnel) will work on the HEPA units to ensure standardized appearance with tape and active or inactive filter setup. They will not assess outcomes.

##### Study intervention compliance

Families will be instructed to place kW meters on the units at the onset of installation within the home to assess usage compliance with HEPA/control units. These meters enable assessment of power consumption and estimate corresponding costs for energy usage. Actual kWh usage during the intervention period will be compared to the usage predicted from laboratory tests. The actual kWh used will be divided by the predicted usage and this quantity multiplied by 100 to determine adherence. Weekly surveys will also include prompts for parent(s)/guardian(s) to report whether they used the HEPA/control unit that week and on what setting it was most commonly used. Note that the kW meters require three-prong outlets. If a home has only two-prong outlets, they will not use the kW meter.

##### Concomitant therapy

No concomitant medications are prohibited.

### Study intervention discontinuation and participant discontinuation/withdrawal

#### Discontinuation of study intervention

If the family/participant chooses not to continue the study intervention (i.e., chooses to stop using the HEPA unit/control air unit), they may choose to allow their child to remain in the study and complete the remaining study procedures as indicated by the study protocol. Participants parents/guardians will still be compensated for study activities, keep HEPA units, and receive replacement filters at the end of the study, even if they do not complete the intervention. If the study intervention is discontinued, the reason for discontinuation will be documented.

#### Participant discontinuation/withdrawal from the study

A parent/guardian is free to withdraw (at any time) their child from participation in the study (including intervention, data collection, and assessments/surveys) without prejudice to further medical treatment (withdrawal of consent). The parent(s)/guardian(s) of the participant will be asked about the reason(s) and the presence of any AEs. The parent(s)/guardian(s) will be told they do not need to answer any questions they do not want to answer. If a parent/guardian of a participant chooses to withdraw their child from the intervention, they will be asked if they want to continue to in the study assessment procedures, including measurement of air quality, submission of symptom diaries and surveys. If parent/guardian of a participant chooses to withdraw their child from all further participation in the study, then no further study activities or data collection will take place.

Moving to a new residence is not a criterion for discontinuation unless it is no longer feasible for the parent/guardian of the participant to complete the study activities.

An investigator may discontinue or withdraw a participant from the study for the following reason(s): If any AE, or other medical condition or situation occurs such that continued participation in the study would not be in the best interest of the participant as determined by the site investigator and/or data safety monitoring board (DSMB).

The reason for participant discontinuation or withdrawal from the study will be recorded on the appropriate CRF. Participants will not be replaced.

#### Lost to follow-up

The following actions must be taken if a parent/guardian of a participant fails to return or complete study assessments (on behalf of their enrolled child):

- Study staff will attempt to contact the parent/guardian of the participant and obtain the study survey data within 2 business days for weekly surveys and within 7 business days for quality of life surveys. They will also counsel the parent/guardian of the participant on the importance of maintaining the assigned study activity schedule on behalf of their enrolled child and ascertain if the parent/guardian of the participant wishes to have their child continue in the study.
- Before a participant is deemed lost to follow-up, the investigator or designee will make every effort to regain contact with the parent/guardian of the participant (at least three telephone calls, and, if necessary, a certified letter to the last known mailing address of the parent/guardian of the participant). These contact attempts will be documented in the participant’s study file.
- Should the participant’s parent/guardian continue to be unreachable, he or she will be considered to have withdrawn from the study with a primary reason of lost to follow-up.

### Study assessments and procedures

#### Efficacy assessments

##### Collection of participant characteristics and risk factors for recurrent wheeze

History will be obtained at the start and end of the study to ensure randomization provides similar characteristics of the intervention and control groups. If there is an imbalance between arms, we will control for these factors in the primary analysis.

##### Participant characteristics and risk factors for recurrent wheeze collect information at baseline or intervention start (by parental report unless otherwise indicated)

- Age (in months) at initial hospitalization for bronchiolitis (medical record review)
- Gestational age at birth (parental report or medical record review)
- Sex/gender (parental report or medical record review)
- Race/ethnicity (parental report or medical record review)
- Parental education
- U.S. Census Tract Rural/Urban (RUCA code) based on residential address
- Viral test results (first hospital admission for bronchiolitis) if available per standard of care testing (medical record review)
- Season of hospitalization for bronchiolitis (medical record review)
- Highest level of respiratory support during bronchiolitis admission (medical record review)
- History of previous wheezing with illness
- Family history of asthma
- Wood stove use in the home (and whether this is the primary heat source)
- Central air conditioning in the home
- Type of cooking stove in the home
- Presence of hood above cooking stove in home
- Use of hood while cooking
- Risk of higher frequency of viral exposures
  - ◦ Daycare attendance
  - ◦ Number of children in home
  - ◦ Number of children in home in daycare or school
  - ◦ Household crowding
- Presence of plumbed (running) water
- Furry pets in the home
- Baseline weekly average PM2.5 home measurements (Purple Air Monitor data report)
- Atopic dermatitis
- Chronic use of asthma medications preceding bronchiolitis hospitalization
- Use of asthma medications with illness preceding bronchiolitis hospitalization
- Received systemic steroid during hospitalization for bronchiolitis (medical record review)
- Square footage of rooms containing HEPA units (during intervention set-up)

##### Collect information at 26 weeks (end of 24-week intervention period) by parental report unless otherwise indicated

- Smokers who live in the home
- Chronic use of asthma medications
- New prescriptions for asthma medications or antibiotics with healthcare visits for respiratory symptoms (determined from weekly survey entries)
- Atopic dermatitis
- Immunization status
- Average number of nights per week away from home (calculated from study data)
- Average number of days per week where the child was away from home more than 6 h (calculated from study data)
- Weekly outdoor PM2.5 concentration (These data may be obtained after the 26-week study period)

##### Air quality measurements (both intervention and control groups)

Family-collected with study team support remotely:

- *Continuous PM Assessment:* Families will install PurpleAir PA-II-SD continuous sensors in the common room and child’s sleep space. The sensor measures numerous environmental factors, but PM2.5 is of primary interest. The PurpleAir is 3.5 × 3.5 × 5 inches and weighs 25 oz with the power supply. The distance from the filtration unit to the PurpleAir will be measured by the family with a tape measure and reported to the study team. Study team personnel will schedule a phone call or video meeting for each family with technical support personnel to assist with equipment setup.

Mean weekly indoor PM2.5 concentration, averaged between the two PurpleAirs in the home, is a secondary endpoint in the trial. Baseline PM2.5 will be measured ideally over at least 4 days.

Data from the PurpleAir can be stored and retrieved in two ways, both of which will be used in this study.

- Each PurpleAir is WiFi-enabled. So that the monitor use does not interfere with a family’s WiFi usage, each participating home will be provided with one mobile password-protected hot spot and necessary data. One hot spot is sufficient to serve both PurpleAirs. The PurpleAir monitors will be connected to the hot spot prior to mailing (done centrally prior to receipt of the monitors by individual study sites) to simplify set up of monitors by participating families.
- PurpleAir sensor data can be retrieved from a public website. However, all sensors used in this study will be kept private to protect participant privacy. Data will be retrieved by University of Montana central site personnel from private sensors using a PurpleAir application programming interface (API) key using automated methods. Data retrieval will occur at frequent intervals to ensure the sensor is operating properly. University of Montana central site staff will perform quality checks on the data, and, if issues arise, they will communicate with coordinators and/or families to troubleshoot the problem or opt to rely on data from the security digital card (see below). If needed, staff will provide additional training or a new instrument. Details of data retrieval, quality assurance, adherence, and data analysis are described in coordinator instructions and checklists and the Data Safety and Monitoring Plan.
- Each PurpleAir is equipped with a security digital (SD) card that logs PM2.5 data in the event of WiFi interruptions. The SD card has sufficient storage to hold at least 6 months of PM2.5 data so that it does not need to be changed during follow-up. The family will mail the PurpleAirs back to the ISPCTN site (or designated central site, i.e., the University of Montana) at the end of follow-up. Research staff will then remove the SD card and download the data. Data obtained through WiFi are considered primary; however, data from the SD card will be used to infill any missing observations. The University of Montana central site team will provide final summary PM2.5 metrics to the DCOC.

##### Weekly survey collection of child’s daily symptoms, healthcare utilization data, time away from home, and study equipment use

The parent(s)/guardian(s) will submit a brief weekly online survey (via the EDC platform) that they receive via text or email. The alternative is a scheduled phone call with a standard script with the study coordinator or other qualified personnel from the study site research team. Parent(s)/guardian(s) of participants will receive a text or email reminder to complete the survey if it is not completed within 1 day of original due date. If the weekly survey is not completed within 1 day of the reminder, the research team will call the participant parent/guardian (ideally within 1 business day) to obtain the survey data by phone or prompt the participant’s parent/guardian to submit it electronically.

The weekly survey will capture 4 elements: (1) the child’s symptoms for the week, (2) number of healthcare visits, (3) time away from home, (4) study equipment use.

Each family will receive a visual tool (paper form named “Symptom Recall Tool”) with the written questions as a prompt/reminder of what will be asked. The family will receive a booklet with a page of the Symptom Recall Tool for each week of the study. The Symptom Recall Tool is an aid for reporting and will not be collected. We estimate that the weekly survey will take 1–5 min to complete electronically.

- Symptom survey: The survey captures three daytime symptoms (cough, wheeze, trouble breathing) and nighttime awakenings due to cough. The responses to the symptom survey in the EDC will be used to determine SFDs. The questions have been used previously to measure SFDs after bronchiolitis over a similar time frame of approximately 6 months in a study testing whether an intervention reduces post-bronchiolitis symptomatic days [5]. The questions are based on the Bronchiolitis Caregiver Diary, a validated measurement tool for respiratory symptoms after acute bronchiolitis [37]. These questions have been used to follow post-bronchiolitis symptoms over a 20-week period, similar to this study [5]. The survey will ask whether the child had any cough, wheeze, or trouble breathing this week (Y/N).
- If No, they will move on to the next section for healthcare visits.
- If Yes, they will receive a prompt for each day to respond Y/N for the presence of the symptom and if there are symptoms present, the survey will ask if any medications were used for respiratory symptoms (family to list names of medications).
- Healthcare visits: The parent/guardian will record the number of hospitalizations, ED/UC visits, and other medical visits for respiratory symptoms. This will be a (Y/N) for whether they participant (child) had a healthcare visit in the past week. If Y, there will be a prompt to enter the number of visits for each visit type. If N, they will move on to the next section, “Time away from home.”
- Time away from home:
  - ◦ Days away from home: The parent/guardian will be prompted to enter how many days that week the child spent more than 6 h outside the home (0–7 days).
  - ◦ Nights away from home: The parent/guardian will be prompted to enter how many nights that week the child spent away from their primary residence (away for vacation, staying with someone else, etc.) (0–7 days).
- Equipment use:
  - ◦ HEPA/control unit use: For each room with a HEPA/control unit, the parent/guardian will be asked to respond Yes/No to whether they used unit and asked to report the usual setting used (1, 2, 3, or 4). If applicable, the parent(s)/guardian(s) will also record the numerical reading visible on the kW meter attached to each HEPA/control unit. This will be a simple entry of two numbers to respond to the survey prompt.
  - ◦ PurpleAir monitor: The parent/guardian will be asked whether the monitor is plugged in with the light on (Yes or No). They will also be asked whether the hot spot is on with bar light and 3 lighted dots on (Yes or No).

##### Quality of life

- The PedsQLTM Pediatric Quality of Life Inventory Infant Scales [99] questionnaire will be administered to a parent/guardian at the end of the intervention period. This is a validated outcome measure of QOL for infants 1–12 months (36 items) and 13–24 months (45 items). The constructs include 5 subdomains: physical functioning, physical symptoms, emotional functioning, and cognitive functioning. The questionnaire will be administered online. The alternative will be for the research coordinator to obtain responses verbally.

##### HEPA unit adherence monitoring

Adherence to HEPA filtration unit use will be monitored by a kW meter attached to each HEPA device (Intertek KILL A WATT® EZ Model P4460.01). Families will be instructed to attach kW meters to the HEPA/control units at the intervention onset. These meters enable assessment of power consumption and estimate corresponding costs for energy usage. Actual kWh usage during the intervention period will be compared to the usage predicted from laboratory tests. The actual kWh used as reported by participants will be divided by the predicted usage and this quantity multiplied by 100 to determine participant adherence to the intervention. Parents/guardians of participants will report the reading from the kW meter (number visible on the screen) on the weekly survey. In addition to measuring use of the HEPA unit for the analysis, study staff will be able to verify that the reading is increasing over time. These data will alert the study team to potential non-usage of the HEPA/control unit or problems with the kW meter to allow troubleshooting.

#### Safety and other assessments

This is a minimal risk study. No changes to standard of care therapies and standard medical treatment will be made for participants based on research data. Children will not receive medical care from the research team and will receive their usual care (such as from their primary care provider). Childproofing is necessary for all equipment to prevent injury to young children, and steps to determine family needs around childproofing will be outlined in the MOP. If circumstances arise such as a significant air pollution exposure that is expected to be prolonged (e.g., major wildfire with air quality impacts in the area) or other conditions where it is medically recommended by the child’s health provider to use active HEPA filtration, the participant may be unmasked.

#### Adverse events and serious adverse events

##### Definition of adverse events (AE)

An AE is any untoward or unfavorable medical occurrence in a human subject, including any abnormal sign, symptom, or disease, temporally associated with the volunteer’s participation in research, whether or not it is considered related to the research intervention. Stable chronic conditions that are present prior to enrollment and do not worsen are not considered AEs and will be accounted for in the subject’s medical history. Exacerbation or worsening of pre-existing conditions are defined as AEs. Each AE will be classified by the investigator as serious (SAE) or nonserious. All AEs will be evaluated for severity, action taken, seriousness, outcome, and relationship to the study intervention. Information on protocol-specific AEs, severe AEs, and SAEs will be collected at scheduled visits and interval phone calls, if needed. Protocol-specific AEs, severe AEs, and SAEs will be collected for the duration of the study. We will only record and track severe AEs, SAEs, and AEs associated with the study intervention and/or study equipment.

##### Definition of serious adverse events (SAE)

An AE or suspected adverse reaction is considered “serious” if, in the view of either the investigator or sponsor, it results in any of the following outcomes:

- Death
- A life-threatening adverse event
- Inpatient hospitalization or prolongation of existing hospitalization
- Persistent or significant incapacity or substantial disruption of the ability to conduct normal life functions
- Important medical events that may not result in death, be life-threatening, or require hospitalization may be considered serious when, based upon appropriate medical judgment, they may jeopardize the participant and may require medical or surgical intervention to prevent one of the outcomes listed in this definition

##### Classification of an adverse event

**Severity of event**

For AEs, the following guidelines will be used to describe severity.

- Mild—Events require minimal or no treatment and do not interfere with the participant’s daily activities.
- Moderate—Events result in a low level of inconvenience or concern with the therapeutic measures. Moderate events may cause some interference with functioning.
- Severe—Events interrupt a participant’s usual daily activity and may require systemic drug therapy or other treatment. Severe events are usually potentially life-threatening or incapacitating. Of note, the term “severe” does not necessarily equate to “serious.”

**Relationship to study intervention**

All AEs must have their relationship to study intervention assessed by the investigator or qualified clinician who evaluates the participant based on temporal relationship and their clinical judgment. The degree of certainty about causality will be graded using the categories below.

- Definitely related—There is clear evidence to suggest a causal relationship, and other possible contributing factors can be ruled out. The clinical event, including an abnormal laboratory test result, occurs in a plausible time relationship to study intervention administration and cannot be explained by concurrent disease or other drugs or chemicals. The response to withdrawal of the study intervention (dechallenge) should be clinically plausible. The event must be pharmacologically or phenomenologically definitive, with use of a satisfactory rechallenge procedure if necessary.
- Probably related—There is evidence to suggest a causal relationship, and the influence of other factors is unlikely. The clinical event, including an abnormal laboratory test result, occurs within a reasonable time after administration of the study intervention, is unlikely to be attributed to concurrent disease or other drugs or chemicals, and follows a clinically reasonable response on withdrawal (dechallenge). Rechallenge information is not required to fulfill this definition.
- Potentially related—There is some evidence to suggest a causal relationship (e.g., the event occurred within a reasonable time after administration of the intervention). However, other factors may have contributed to the event (e.g., the participant’s clinical condition, other concomitant events). Although an AE may rate only as “possibly related” soon after discovery, it can be flagged as requiring more information and later be upgraded to “probably related” or “definitely related”, as appropriate.
- Unlikely to be related—A clinical event, including an abnormal laboratory test result, whose temporal relationship to study intervention administration makes a causal relationship improbable (e.g., the event did not occur within a reasonable time after administration of the study intervention) and in which other drugs or chemicals or underlying disease provides plausible explanations (e.g., the participant’s clinical condition, other concomitant treatments).
- Not related—The AE is completely independent of study intervention administration, and/or evidence exists that the event is definitely related to another etiology. There must be an alternative, definitive etiology documented by the clinician.

**Expectedness**

The site investigator or qualified clinician designee will be responsible for determining whether a severe AE or SAE is expected or unexpected. A severe AE or SAE will be considered unexpected if the nature, severity, or frequency of the event is not consistent with risks of underlying chronic medical conditions, is not an event measured in study data collection (hospitalization, ED visit, etc.), or is not related to function of the study intervention equipment.

Potential expected events could include the following:

- Cough
- Wheeze
- Trouble breathing
- Medical or emergency department/urgent care visit for respiratory complaint

**Time period and frequency for event assessment and follow-up**

All potentially related, severe AEs, and SAEs will be recorded on the appropriate CRF. Information to be collected includes event description, approximate date of onset, clinician’s assessment of severity, relationship to study intervention (assessed by site investigator or qualified clinician designee), and date of the resolution/stabilization of the event. SAEs occurring while on the study must be documented appropriately regardless of relationship, and must be followed until one of the following criteria is met: study completion, resolution, the condition stabilizes, the event is otherwise explained or is judged by the site investigator or qualified clinician designee to be no longer clinically significant, or the participant is lost to follow-up.

Any medical condition that is present at the time that the participant is screened will be considered as baseline and not reported as an AE. However, at the onset of the intervention and anytime during the study if the study participant’s condition deteriorates and meets the definition of severe AE, SAE, or is possibly related to the study intervention, it will be recorded as an AE. Changes in the severity of an AE will be documented to allow an assessment of the duration of the event at each level of severity to be performed. AEs characterized as intermittent require documentation of onset and duration of each individual episode.

The site investigator or qualified designee will record all potentially related, severe AEs, and SAEs with start dates occurring any time after informed consent is obtained until 30 days after the last day of study participation. At each study visit or interval phone call, the investigator will inquire about the occurrence of potentially related, severe AEs, and SAEs since the last visit. Events will be followed for outcome information until resolution or stabilization.

**Adverse event reporting**

All potentially related or severe AEs that occur after informed consent is obtained until the last day of study participation will be documented in the participant’s source documents and in the AE section of the CRF.

**Serious adverse event reporting**

The site investigator or qualified delegate will immediately report to the appropriate entities (which may include the DCOC, NIH, DSMB, and local and/or reviewing IRBs) any SAE, when required to do so based on that entity’s policies and procedures. Reports need to include the information required by the entities’ policies and procedures. Study endpoints that are serious adverse events (e.g., hospitalization) must be reported in accordance with the protocol unless there is evidence suggesting a causal relationship between the study intervention and the event (e.g., death from anaphylaxis). In that case, the investigator must immediately report the event.

All SAEs will be followed until satisfactory resolution or until the site investigator deems the event to be chronic or the participant is stable. Other supporting documentation of the event may be requested by the DCOC and should be provided as soon as possible.

**Reporting events to participants**

The parent(s)/guardians of participants will be notified of those study-related (or potentially study-related) SAEs or unanticipated problems (UPs) that may affect either the parent/guardian’s willingness to allow their child to continue with the study or the future health of the participant. This determination can be made by any of the following: the reviewing IRB, the medical monitor, the DSMB, the DCOC, or the NIH. The person or oversight body that makes the determination will inform the DCOC, which will instruct the site PIs/study coordinators to contact those participants enrolled through their site. Contacts with parent(s)/guardian(s) of participants, if necessary, will be recorded on the appropriate CRF and/or study log.

##### Unanticipated problems

**Definition of unanticipated problems (UPIRTSOS)**

Unanticipated problems (UPs or UPIRTSOs) involving risks to participants or others include, in general, any incident, experience, or outcome that meets all of the following criteria:

- Unexpected in terms of nature, severity, or frequency given (a) the research procedures that are described in the protocol-related documents, such as the Institutional Review Board (IRB)-approved research protocol and informed consent document and (b) the characteristics of the participant population being studied;
- Related or possibly related to participation in the research (“possibly related” means there is a reasonable possibility that the incident, experience, or outcome may have been caused by the procedures involved in the research); and
- Suggests that the research places participants or others at a greater risk of harm (including physical, psychological, economic, or social harm) than was previously known or recognized.

## Unanticipated problem reporting

The investigator will report UPS (UPIRTSOs)—and potential UPIRTSOs—to the DCOC (who will in turn report to the reviewing IRB when the reviewing IRB is UAMS) and the local IRB as well as any other persons or groups noted in the DSMB charter. Reports to NIH and the DSMB will be made by following the chain of notification.

These problems must be reported to the reviewing/local IRBs according to the reviewing/local IRB’s contemporaneous policies and procedures.

For most sites involved in the study, the reviewing IRB will be the University of Arkansas for Medical Sciences (UAMS) IRB. The UAMS IRB contemporaneous policy, 10.2., *Events that must be reported to the IRB and IRB Actions (effective July 6, 2020)* is available via https://irb.uams.edu/irb-policies/current-irb-policies/

Reports typically require the following information:

- Protocol identifying information: protocol title and number, PI’s name, and the IRB project number
- Event date
- Event location
- Nature of the risk
- How the risk relates to research
- A detailed description of the event, incident, experience, or outcome
- A description of any changes to the protocol or other corrective actions that have been taken or are proposed in response to the UP

Examples of UPs/UPIRTSOs that are not an AE or SAE, but which would need to be reported include:

- Breach of confidentiality
- Manufacturer recall of equipment used in the protocol

**Reporting unanticipated problems to participants**

The parent(s)/guardian(s) of participants will be notified of those study-related (or potentially study-related) SAEs or UPs that may affect either the parent(s)/guardian(s) willingness to allow their child to continue with the study or the future health of the participant. This determination can be made by any of the following: the IRB, the medical monitor, the DSMB, the DCOC, or the NIH. The person or oversight body that makes the determination will inform the DCOC, which will instruct the site PIs/study coordinators to contact those participants enrolled through their site. Contacts with parent(s)/guardian(s) of participants, if necessary, will be recorded on the appropriate CRF and/or study log.

### Statistical considerations

#### Statistical hypotheses

Primary objective: To test the efficacy of a HEPA filtration unit home intervention, relative to the control arm, with respect to respiratory symptom burden (as measured by symptom-free days; SFD) over 24 weeks following activation of filtration.

Primary endpoint: Number of caregiver-reported SFDs over 24 weeks following activation of filtration (SFD defined as a 24-h period without coughing, wheezing, or trouble breathing).

Statistical hypothesis: Mean of SFDs in the HEPA filtration home intervention group is larger than mean of SFDs in the control group.

Secondary objective 1: To test the efficacy of a HEPA filtration home intervention, relative to the control arm, on the number of unscheduled healthcare visits for respiratory symptoms over 24 weeks following activation of filtration.

Secondary endpoint 1: Caregiver-reported counts of unscheduled healthcare visits from each of the metrics including hospitalizations, emergency department (ED) visits, urgent care (UC) visits, and other unscheduled medical visits for respiratory complaints (cough, wheeze, or trouble breathing). A sum of counts (or total counts) of all metrics is also used as the secondary endpoint.

Statistical hypothesis 1: Mean of counts of unscheduled healthcare visits for respiratory symptoms in the intervention group is smaller than mean of counts of unscheduled healthcare visits for respiratory symptoms in the control group.

Secondary objective 2: To test the efficacy of HEPA filtration home intervention, relative to the control arm, on difference in QOL.

Secondary endpoint 2: Total QOL score, as measured by the PedsQLTM Pediatric Quality of Life Inventory Infants Scales questionnaire at the end of the intervention period.

Hypothesis 2: Child QOL will be higher in families that receive the HEPA intervention compared to controls.

Secondary objective 3: To test the efficacy of HEPA filtration home intervention, relative to the control arm, on PM2.5 levels in the home over 24 weeks following activation of filtration.

Secondary endpoint 3: Average PM2.5 levels as measured by 2 in-home PurpleAir monitors over 24 weeks and scaled to the unit of μg/m^3^ per week.

Hypothesis 3: Mean of PM2.5 level in the intervention group is lower than mean of PM2.5 level in the control group.

#### Sample size determination

##### Sample size justification

We plan to enroll 228 participants, or 109 participants per arm. To account for an anticipated attrition rate of 10% per arm, the power analysis is based upon a sample size of 196 participants, or 98 participants per arm. From a similar previously published study, it was found the mean of days with symptoms was 70 days (equivalently the mean of SFDs was 98 days out of total 24 weeks or 168 days of observation), and the standard deviation was 43 days.5 The proposed sample size will provide 90% power to detect an effect size of 0.465, or a difference of 20 symptom-free days with a standard deviation of 43 days, using a two sample *t*-test.

##### Randomization scheme

The randomization will be stratified by site. Within each site, participants will be randomized in a 1:1 allocation to receive active HEPA filtration (intervention group) and inactive HEPA unit (control group). Permuted block randomization will be employed.

#### Populations for analyses

##### Analysis population

Intent-to-treat (ITT) population: The ITT population will include all participants who are randomized to either HEPA filtration (intervention group) or inactive filter unit (control group) (referred to as two groups in the analysis section).

Per-protocol (PP) population: The PP population will include all participants who are randomized to either the HEPA filter (intervention group) or inactive filter (control group) and have used HEPA unit on average for both units > 80% of the time.

The primary set of analyses for this study will be based on ITT population. A separate analysis will be done with the PP population.

#### Statistical analyses

##### General approach

- Descriptive statistics: All numerical variables will be summarized using mean ± standard deviation and median (minimum, maximum). All categorical variables will be summarized using frequency (in %).
- Inference tests: All proposed statistical tests are two-sided. A *p*-value < 0.05 is considered statistically significant.
- Covariates: Covariates will be compared between groups (intervention vs. control) using two sample *t*-tests, or Wilcoxon rank sum tests if they are continuous variables, and chi-square tests or Fisher’s exact tests if they are categorical variables. Each of the continuous covariate variables will be assessed of its correlation to the primary endpoint, or each of the secondary endpoints using Pearson’s correlation coefficient or Spearman’s correlation coefficient. Similarly, each of the categorical covariate variables will be assessed of its association to the primary endpoint, or each of the secondary endpoints using an ANOVA model, or a Kruskal–Wallis test. A covariate showing a significant association to intervention, a significant correlation or association to the primary (or secondary) endpoint will be considered as the adjusting (controlling) covariate and will be added as adjusting independent variable in the statistical models proposed for primary and secondary analyses.
- Model assumptions: Primary and secondary endpoints will be inspected of normal distribution assumptions using the histogram plots. If the variable is noticeably right (or seldomly left) skewed, then a transformation variable will be used in the parametric models to ensure the assumption of normality is met. As an alternative approach, a generalized linear model will be proposed to the variable using a distribution assumption fitting the data properly.

#### Analysis of the primary efficacy endpoint

- Primary objective: To test the efficacy of a HEPA filtration unit home intervention, relative to the control arm, with respect to respiratory symptom burden (as measured by symptom-free days; SFD) over 24 weeks following activation of filtration.
- Primary endpoint: Number of caregiver-reported SFDs over 24 weeks following activation of filtration (SFD defined as a 24-h period without coughing, wheezing, or trouble breathing).
- Statistical hypothesis: Mean of SFDs in the HEPA filtration home intervention group is larger than mean of SFDs in the control group. The hypothesis testing is the comparison of superiority.
- Statistical procedures: The hypothesis will be tested using a mixed effect model after accounting for within cluster correlation. The model uses the primary endpoint as the dependent variable, and the intervention effect (intervention vs. control) as the independent variable or the fixed effect with site as a random effect.
- Missing data: The primary endpoint will be imputed if there is any missing observation in the ITT. The statistician will assess the missing patterns to determine if the cause of missing is missing at random (MAR), missing completely at random (MCAR), or missing not at random (MNAR). Imputation methods such as multiple imputation (MI) methods and pattern-mixture methods will be used in imputation and analyses.

#### Analysis of the secondary endpoints

- Secondary endpoint 1: Caregiver-reported unscheduled healthcare visits. The proposed statistical method will be a generalized mixed effect model. The dependent variable will be counts of unscheduled health care visits from each of the metrics or the sum of all metrics. Each variable of counts is considered to follow a negative binomial distribution and its log link will be used to connect the independent variable, or the fixed effect of intervention effect with site as a random effect.
- Secondary endpoint 2: Total PedsQLTM Infant Scales score. The proposed statistical model will be a mixed effect model using Total PedsQL score as the dependent variable, the intervention effect as the fixed effect, with site as a random effect. Means (and SEs) of Total PedsQL score estimated from the mixed effect model will be presented in the final result and compared between groups through a *p*-value to reach a statistical conclusion of significance and superiority.
- Secondary endpoint 3: PM2.5 levels. The proposed statistical model will be a mixed effect model using PM2.5 level as the dependent variable, and the intervention effect as the fixed effect, and site as a random effect. Means and SEs of PM2.5 level from the mixed effect model will be presented in the final result. A *p*-value of the difference of means between groups will be used to reach a statistical conclusion of significance and superiority.
- Missing data: Missing data of secondary endpoint will be assessed of causes of missing and imputed in analyses, following the same methods proposed for the primary endpoint.

#### Safety analyses

Safety analysis: Any AE related to the study groups specific to child participant will be documented and summarized as overall and by study groups using aforementioned descriptive statistics. Safety analysis and reports will be made as specified in the Data Safety and Monitoring Plan (DSMP).

#### Planned interim analyses

Interim analysis: An interim efficacy analysis has been planned for this study when 50% of study participants (49 participants in both groups) have completed the follow-up period. We will employ Lan & DeMets’ alpha-spending function together with O’Brien-Fleming boundaries to preserve the overall type I error rate at 0.05 and power at 90% in the final analysis. The boundaries and operating characteristics for the proposed analyses are provided in the table below. In the event that findings from interim analysis provide evidence in favor of futility, the study team may consider halting the study.

**Table:** 

Analysis	Information fraction	Reject H0 (efficacy)	Overall α spent	Reject H1 (futility)	Overall β spent
Interim	0.50	|*zz*|> 2.963	0.0003	|*zz*|< 0.200	0.012
Final	1.00	|*zz*|> 1.969	0.05	|*zz*|< 1.969	0.102

#### Sub-group analyses Page 55 line 1264–1267

A stratified analysis of primary endpoint and secondary endpoints will be done: (a) by site among those sites with ≥ 10 participants per study groups, (b) by sex for those levels/categories of sex with ≥ 10 participants, and (c) race-ethnicity for those levels/categories of race-ethnicity with ≥ 10 participants.

### Supporting documentation and operational considerations

#### Regulatory, ethical, and study oversight considerations

##### Informed consent process

Consent/assent and other informational documents provided to participants.

Completed (all signatures affixed) written consent forms, approved by the reviewing IRB and describing the study intervention, study procedures, and risks, will be given to the participant’s parent(s)/legal guardian prior to starting study intervention.

##### Consent procedures and documentation

Informed consent is a process that starts before the individual agrees to participate in the study (or allows their child to be a participant) and continues throughout the individual’s study participation. For sites (i.e., all sites except Native American sites) using the UAMS IRB as their reviewing IRB, the contemporaneous version of UAMS IRB policy 15.5, *Informed Consent Process*, is applicable and must be followed. The policy is available at https://irb.uams.edu/irb-policies/current-irb-policies/. If there are any discrepancies between this protocol and applicable reviewing IRB polici(es), the more stringent requirements apply.

The written informed consent form will generally be signed during the child’s hospitalization. Consent may also be obtained in the 7 days after hospitalization provided all study enrollment, randomization, and intervention set-up procedures can still be completed within 7 days post-hospitalization. The consent form, including site-specific local context, will be IRB-approved and the parent or legal guardian of the potential participant will be asked to read and review the document. The site investigator or qualified delegate will explain the research study in language that the parent/legal guardian of the participant can understand and will answer any questions that may arise. The explanation will include the purposes, procedures, and potential risks of the study and include the rights of their child as a research participant. Parents/legal guardians of participants must be informed that participation is voluntary and that they may withdraw their child from the study at any time, without prejudice. The rights and welfare of the participants will be protected by emphasizing to parents/guardians of participants that the quality of their child’s medical care will not be adversely affected if they do not allow their child to participate in this study.

Parents/legal guardians of participants will be given the opportunity to carefully review the written consent form and ask questions prior to signing. The parent/guardian of the potential participants must also be given the opportunity to discuss the study with their family or surrogates or think about it prior to agreeing to allow their child to be a participant. The parent/legal guardian of the participant must sign the informed consent form prior to any study-specific procedures being done. A copy of the informed consent form (ICF), signed by all parties—including the person obtaining consent, will be given to the parent/guardian of the participants for their records.

Due to the age (< 2 years old) of the children participating in the study, assent will not be obtained.

The investigator or qualified delegate will document the processes in the source documents (research or medical record of the participant). Consent documentation will include, at a minimum:

1. The title of the trial,
2. The date the participant entered into the trial,
3. The name of the site investigator,
4. The name of the person(s) obtaining the informed consent, and
5. A statement that the parent/legal guardian of the participant received a copy of the signed form.

The following additional documentation is recommended, but it is not required:

1. A list of who else was present during the process,
2. The type of questions asked by the parent/guardian of the participant,
3. A summary of details that demonstrate the parent/guardian of the participant understood the information, and
4. A description other specific details related to that case.

##### Remote consenting

If needed, remote consenting may be used to enroll a participant after the child has left the hospital. All communications will be done via HIPAA-compliant methods such as telephone, personal delivery of documents, US postal service, REDCap, or other compliant electronic platform. The remote consent process will parallel the consent processed used for in-person consenting. The only difference will be the method(s) of communication. The study team will ensure that, as with in-person consenting, the parent/legal guardian of the participant is given sufficient opportunity to ask questions, is able to understand the nature of this study and what participation entails, and is provided a copy of the final, completed consent signed by all parties involved, including the research team member who obtained consent and, when applicable, the site investigator. This final, signed consent will be provided via a HIPAA-compliant method or a method that the parent/legal guardian of the participant has agreed to in writing. The site research team members working on the consenting process will ensure that any parent/legal guardian who is consenting remotely has the authority to consent for the child.

##### Study discontinuation and closure

This study may be temporarily suspended or prematurely terminated if there is sufficient reasonable cause. Written notification, documenting the reason for study suspension or termination, will be provided by the suspending or terminating party to parents/guardians of study participants, investigator, funding agency, sponsor, and regulatory authorities. If the study is prematurely terminated or suspended, the PI will promptly inform parents/guardians of study participants, the IRB, and sponsor and will provide the reason(s) for the termination or suspension. Parents/guardians of study participants will be contacted, as applicable, and be informed of changes to study visit schedule.

Circumstances that may warrant termination or suspension include, but are not limited to:

- Determination of unexpected, significant, or unacceptable risk to participants
- Insufficient compliance to protocol requirements
- Data that are not sufficiently complete and/or evaluable
- Evidence of study futility of the primary endpoint

If the study is temporarily suspended, it may resume once concerns about safety, protocol compliance, and data quality are addressed, and satisfy the sponsor, DSMB, and IRB

##### Confidentiality and privacy

Participant confidentiality and privacy is strictly held in trust by the participating investigators, their staff, and the sponsor(s) and their interventions. Therefore, the study protocol, documentation, data, and all other information generated will be held in strict confidence. No information concerning the study or the data will be released to any unauthorized third party without prior written approval of the sponsor.

All research activities will be conducted in as private a setting as possible.

The study monitor, other authorized representatives of the sponsor, representatives of the IRB, or regulatory agencies may inspect all documents and records required to be maintained by the investigator, including but not limited to medical records (office, clinic, or hospital) for the participants in this study. The clinical study site will permit access to such records.

The study participant’s contact information (i.e., contact information of parent/guardian of participant) will be securely stored at each clinical site for internal use during the study. At the end of the study, all records will continue to be kept in a secure location for as long a period as dictated by the reviewing IRB, Institutional policies, or sponsor requirements.

Study participant research data, which is for purposes of statistical analysis and scientific reporting, will be transmitted to and stored at the DCOC. This will not include the participant’s parents/guardians contact or identifying information. Rather, individual participants and their research data will be identified by a unique study identification number. The study data entry and study management systems used by clinical sites and by DCOC research staff will be secured and password protected. At the end of the study, all study databases will be de-identified and archived at the DCOC.

##### Multi-site communications (IRB-related)

This study will conducted at various sites (approximately 17 hospitals) within the ISCPTN network. All sites, except the Native American site in Alaska, will cede to the UAMS IRB as the reviewing IRB (per SMART IRB definitions). The study-specific IRB-related communications plan was constructed from the SMART IRB template and uses SMART IRB recommendations for communications. This plan will be submitted to the IRB as a separate study-specific document. The DCOC will serve as the lead study team and will be the intermediary between the sites and the UAMS IRB as the central (or single) IRB (i.e., cIRB). Other types of communications (i.e., related to data, study deviations) between DCOC and the sites are detailed in their respective appropriate sections of this protocol.

##### Future use of stored specimens and data

Data collected for this study will be analyzed and stored at the DCOC. Permission to transmit data to the DCOC will be included in the informed consent.

No specimens will be collected or stored for this trial. Regarding stored data, study personnel will document all trial interactions, and these will be password protected in a secured facility/location.

The study team will place participant’s de-identified data and other limited information, such as race and ethnic group, into one or more centralized database(s). The study team will share this data in compliance with the ISPCTN and NIH data sharing policies.

For future studies using any procedures or analysis not specified in this protocol, IRB approval is required. In the event that another investigator/collaborator has a meaningful purpose for accessing the data retrieved in this protocol, the DCOC must consult the PIs and the IRB must approve.

##### Safety oversight

Safety oversight will be under the direction of a DSMB composed of individuals with the appropriate expertise, including pediatrics, environmental health, and biostatistics. Members of the DSMB will be independent from the study conduct and free of conflict of interest, or measures will be in place to minimize perceived conflict of interest. The DSMB will meet on a regular basis, per the DSMB charter, to assess safety and efficacy data of the study. The DSMB will operate under the rules of an approved charter. Data elements that the DSMB needs to assess are defined in the charter. The DSMB will provide its input to the NIH and the sponsor.

The role of the Medical Monitor is to provide input on safety considerations, evaluate safety trends, and provide oversight throughout the life cycle of the clinical research, in accordance with the approved protocol. This role includes review and monitoring of safety events on a regular basis, advising the protocol investigators on trial-related medical questions or problems, as needed, and to review cumulative participant safety data and make recommendations regarding the data to the DSMB. The Medical Monitor will remain blinded to treatment assignment during safety event review, unless unblinding is warranted to optimize management of an adverse event or for other safety reasons.

##### Clinical monitoring

Clinical site monitoring will be conducted to ensure that the rights and well-being of trial participants are protected, that the reported trial data are accurate, complete, and verifiable, and that the conduct of the trial complies with the current approved protocol and other IRB-approved documents, with ICH E6(R2), with applicable regulatory requirement(s) and other documents, including the study-specific Manual of Procedures (MOP), needed to complete study conduct.

Monitoring for this study will be performed by a member of the DCOC staff or their designee.

Monitoring will be planned to be conducted on site, or remotely, according to the Site Monitoring Plan. Monitors will use the Site Monitoring Plan to guide their review and guide the documentation of their activities and findings. The Site Monitoring Plan will describe who will conduct the monitoring, the frequency at which monitoring will be done, at what level of detail monitoring will be performed, and provide details for the distribution of monitoring reports.

##### Quality assurance and quality control

Each IRB-approved research site entering data will perform internal quality management of study conduct, data collection, documentation, and completion. Sites that ceded to the UAMS IRB (as central IRB) will follow applicable UAMS IRB policies, available at https://irb.uams.edu/irb-policies/current-irb-policies/. A listing of applicable UAMS IRB policies will be provided in the MOP. If local requirements conflict with UAMS IRB policies, sites will consult with DCOC to help determine which policies and procedures need to be followed. Each site will follow the trial-specific MOP and any applicable site-specific SOPs and/or local (site-specific) IRB policies. The clinics and ECHO ISPCTN site awardees will provide direct access to all their facilities, source data/documents, and reports for the purpose of monitoring or auditing by the DCOC and inspection by local and applicable authorities with oversight responsibilities. When electronic health records are source data/documents, sites must provide read-only access for anyone authorized to inspect or verify records.

Following the applicable monitoring SOPs, the monitors will verify that the clinical trial is conducted and that data are generated, documented (recorded), and reported in compliance with the protocol, the trial-specific Site Performance Plan, site-specific SOPs, the ICH GCP E6(R2), and applicable requirements.

We will implement QC procedures for the database and DCOC-maintained records in accordance with the Site Performance Plan, MOP, data safety monitoring plan (DSMP), and applicable SOPs. We may communicate information about any data anomalies to the sites(s) for clarification/resolution.

We will address issues uncovered during QA, QC, or monitoring activities through simple corrections or root-cause analysis, followed by instituting corrective and preventative action (CAPA), as appropriate and as described in the MOP.

*Data quality assurance*: Each variable will be provided a predefined entering format a range before data entry. Each data entry will be monitored for missing observations and discrepancies based upon predefined variable settings. All missing observations and discrepancies will be flagged and reported to study personnel (investigators and site coordinators) for further investigating the sources of problems. Problems associated to human errors, system errors, and device malfunction at data entry will be corrected following proper steps. All actions will also be recorded for backtracking and future reference. The detailed plans for data quality assurance will be specified in the DSMP.

##### Data handling and record keeping

A formal data management plan will describe and document the data and workflow for the trial. The data management plan and associated documentation will specify all operations performed on data from origination to database lock, including detailed descriptions of source documentation, CRFs, instructions for completing forms, data handling and record keeping procedures, procedures for data monitoring, and reconciliation procedures and coding dictionaries to be used, if applicable. The data management plan will also describe the specific data collection and management responsibilities required of the sponsor, study PI(s), the sites, and the DCOC. The contents of the data management plan will be consistent with those described in the Good Clinical Data Management Practices (GCDMP).

Data collection is the responsibility of the clinical trial staff at the individual ECHO ISPCTN sites under the supervision of the site investigator. The site investigator is responsible for ensuring the accuracy, completeness, legibility, and timeliness of the data reported and will carefully monitor study procedures to protect the safety of research subjects, the quality of the data, and the integrity of the study. All source documents must be completed using standard good documentation practices (i.e., the ALCOA-C method [attributable, legible, contemporaneous, original, accurate, and complete]).

It is best practice for ECHO ISPCTN site coordinators to use hard copies of any data recorded on paper CRFs or trial visit worksheets/assessment forms as source document worksheets recording data for each participant consented in the trial. Study personnel will enter clinical data into an EDC system that complies with HIPAA regulations, provided by the DCOC at UAMS. The EDC system includes password protection and internal quality checks, such as automatic range checks, to identify data that appear inconsistent, incomplete, or inaccurate. Study personnel will enter clinical data directly from the source documents. Data recorded in the EDC derived from source documents must be consistent with the data recorded on the source documents.

##### Study records retention

Throughout the course of the trial, all site awardees and clinics will retain the source documents on site in accordance with current site-specific medical record storage procedures.

Sites must retain all trial documents in accordance with local and/or federal regulations, whichever is most stringent. Sites will not destroy any records without the written consent of the sponsor, if applicable. It is the responsibility of the sponsor to inform all investigators when these documents no longer need to be retained.

##### Protocol and study deviations

A deviation is any instance of failure to follow, intentionally or unintentionally, the requirements of the clinical trial protocol, ICH E6(R2) (i.e., “GCP”), the study-specific MOP, or other documents needed to complete study conduct. The instance of failure may be on the part of the participant, the investigator, or other study staff personnel. When deviations occur, the sponsor, and/or site team(s) will ensure actions are taken to correct the problem and, as needed, prevent the deviation from recurring.

These practices are consistent with ICH E6(R2) (available at https://www.fda.gov/files/drugs/published/E6%28R2%29-Good-Clinical-Practice--Integrated-Addendum-to-ICH-E6%28R1%29.pdf).

Sites must record all deviations in the trial source documents. Whenever a deviation occurs, the DCOC will ensure an appropriate assessment is conducted. The assessment should include documentation of the severity and risk of the deviation. Sites that have a system set up for assessing deviations and doing their own corrections via corrective and preventive action (CAPA) plans will do so according to their site SOPs/system. The site will send copies of their CAPA plan documentation to the DCOC. If a site does not have their own quality assurance system to complete adequate deviation review and assessments, corrections, and CAPA plans, then the DCOC will provide that function for the sites. Details of these processes will be provided in the MOP and/or trial-specific SOPs. Essentially, the site and/or the DCOC will request/ensure that there is either a CAPA plan initiated or a simple one-time correction is performed, as appropriate.

##### Publication and data sharing policy

We will conduct this trial in accordance with the following publication and data sharing policies and regulations:

- NIH Public Access Policy, which ensures that the public has access to the published results of NIH-funded research. It requires scientists to submit final peer-reviewed journal manuscripts that arise from NIH funds to the digital archive PubMed Central upon acceptance for publication.
- ECHO ISPCTN Publications and Presentations Policy, which ensures accurate, responsible, and efficient communication of findings from ECHO ISPCTN clinical trials. The ECHO ISPCTN Steering Committee has approved and ratified the ECHO ISPCTN Publications and Presentations Policy, which includes representatives from all site awardees, as well as representatives from the NIH and the DCOC.
- NIH Data Sharing Policy and the policy on the Dissemination of NIH-Funded Clinical Trial Information and the Clinical Trials Registration and Results Information Submission Rule. We will register this trial at ClinicalTrials.gov, and we will submit trial results to ClinicalTrials.gov. In addition, we will make every attempt to publish results in peer-reviewed journals. Other researchers may request data from this trial by contacting Jeannette Lee, PhD, at the DCOC.

##### Conflict of interest policy

The independence of this trial from any actual or perceived influence, such as by the pharmaceutical industry, is critical. Therefore, we will disclose and manage any actual conflict of interest of persons who have a role in the design, conduct, analysis, publication, or any aspect of this trial. Furthermore, persons who have a perceived conflict of interest will be required to have such conflicts managed in a way that is appropriate to their participation in the design and conduct of this trial. The trial leadership in conjunction with the NIH ECHO office has established policies and procedures for all trial group members to disclose all conflicts of interest and will establish a mechanism for the management of all reported dualities of interest.

## Trial status

Protocol version number and date: V-03, 08–23-2022

ClinicalTrials.gov Identifier: NCT05615870 (list of trial sites available)

**Date recruitment began:** 11/07/2023

When recruitment will be completed: 05/06/2024

## Trial registration—data set

All items from the World Health Organization Trial Registration Data Set

**Table:** 

Data category	Information
Primary registry and trial identifying number	ClinicalTrials.gov NCT05615870
Date of registration in primary registry	14 November, 2022
Secondary identifying numbers	n/a
Source(s) of monetary or material support	National Institutes of Health
Primary sponsor	NIH, Environmental Influences on Child Health Outcomes (ECHO) Program Institutional Development Award (IDeA) States Pediatric Clinical Trials Network
Secondary sponsor(s)	n/a
Contact for public queries	Lora A Lawrence, RN lawrenceloraa@uams.edu
Contact for scientific queries	Jessica Snowden, MD,MS,MHPTT JSnowden@uams.edu
Public title	Bronchiolitis Recovery and the Use of High Efficiency Particulate Air (HEPA) Filters (BREATHE)
Scientific title	Bronchiolitis Recovery and the Use of High Efficiency Particulate Air (HEPA) Filters (The BREATHE Study)
Countries of recruitment	United States
Health condition(s) or problem(s) studied	Bronchiolitis (post-acute bronchiolitis respiratory symptoms)
Intervention(s)	Active comparator: HEPA filtration (Winix 5500–2 HEPA filtration units)
	Placebo comparator: Identical appearing placebo Winix unit without HEPA or carbon filters
Key inclusion and exclusion criteria	Ages eligible for study: < 12 months Sexes eligible for study: both Accepts healthy volunteers: yes
	Inclusion criteria: • Age < 12 months at hospital admission • First-time hospitalization for bronchiolitis • One primary residence (> 5 days per week) • Parent, legal guardian or other legally authorized representative consents to allow their child to participate and agrees to participate in all study activities • Electricity in the home (required to power the study equipment) • Wireless internet access or cellular service access in the home* • English or Spanish-speaking parent or guardian
	Exclusion criteria: • Chronic airway or respiratory conditions requiring home oxygen, mechanical ventilation, or tracheostomy dependence; known immunodeficiency, hemodynamically significant cardiac conditions including those requiring medication or oxygen; cystic fibrosis; neuromuscular disease; eligible for palivizumab (per AAP guidelines87) • Use of stand-alone home HEPA filtration other than study-related HEPA units in the home • Household member who smokes (any type), vapes, or uses e-cigarettes • Intention to move in the next 6 months • Enrolled or plans to enroll in an interventional clinical trial for treatment of acute bronchiolitis or sequelae of bronchiolitis, unless permission given by the PI • Another child in the household is enrolled in this study (one child per household can enroll)
Study type	Interventional
	Allocation: Randomized Intervention model: Parallel assignment Multi-center, parallel, double-blind, randomized controlled clinical trial; triple masking (Participant, Care Provider, Investigator) Framework: Superiority
	Primary purpose: prevention
	Phase: n/a
Date of first enrolment	November 7, 2022
Target sample size	228
Recruitment status	Recruiting
Primary outcome(s)	Determine if use of HEPA filtration units reduces respiratory symptom burden for 24 weeks compared to use of control units
Key secondary outcomes	Assess the efficacy of the HEPA intervention relative to control on 1) number of unscheduled healthcare visits for respiratory complaints, 2) child quality of life, and 3) average PM_2.5_ levels in the home

### Supplementary Information

**Supplementary Material 1. **

## Data Availability

The DCOC (UAMS) and NIH will have access to the final trial dataset and the disclosed contractual agreements with the study site entities. *Publications and data sharing policy* We will conduct this trial in accordance with the following publication and data sharing policies and regulations: *NIH Public Access Policy*, which ensures that the public has access to the published results of NIH-funded research. It requires scientists to submit final peer-reviewed journal manuscripts that arise from NIH funds to the digital archive PubMed Central upon acceptance for publication. *ECHO ISPCTN Publications and Presentations Policy*, which ensures accurate, responsible, and efficient communication of findings from ECHO ISPCTN clinical trials. The ECHO ISPCTN Steering Committee has approved and ratified the ECHO ISPCTN Publications and Presentations Policy, which includes representatives from all site awardees, as well as representatives from the NIH and the DCOC. *NIH Data Sharing Policy and the policy on the Dissemination of NIH-Funded Clinical Trial Information and the Clinical Trials Registration and Results Information Submission Rule*. We will register this trial at ClinicalTrials.gov, and we will submit trial results to ClinicalTrials.gov. In addition, we will make every attempt to publish results in peer-reviewed journals. De-identified data will be shared on the NICHD DASH.

## Abbreviations

AE: Adverse event
API: Application programming interface
CFR: Code of Federal Regulations
CMP: Clinical Monitoring Plan
COPD: Chronic obstructive pulmonary disease
CRF: Case report form
DCOC: Data Coordinating and Operations Center
DSMB: Data Safety Monitoring Board
ECHO: Environmental Influences on Child Health Outcomes
ED: Emergency Department
EDC: Electronic Data Capture
EPA: Environmental Protection Agency
GCP: Good Clinical Practice
HEPA: High Efficiency Particulate Air
HIPAA: Health Insurance Portability and Accountability Act
IAQ: Indoor air quality
ICF: Informed consent form
ICH: International Conference on Harmonisation
IRB: Institutional Review Board
ISPCTN: IDeA States Pediatric Clinical Trials Network
kW: Kilowatt
kWh: Kilowatt hour
LRTI: Lower respiratory tract infection
MOP: Manual of Procedures
NCT: National Clinical Trial
NIH: National Institutes of Health
NO2: Nitrogen dioxide
PI: Principal Investigator
PedsQL^TM^: Pediatric Quality of Life Inventory
PM: Particulate matter
PM_2.5_: Particulate matter < 2.5 µm in diameter
QA: Quality assurance
QC: Quality control
QOL: Quality of life
RCT: Randomized controlled trial
RSV: Respiratory syncytial virus
RUCA: Rural-Urban Commuting Area
RV: Rhinovirus
SAE: Serious adverse event
SAP: Statistical analysis plan
SD: Secure digital
SFD: Symptom-free days
SMART IRB: A platform designed to ease common challenges associated with initiating multisite research and to provide a roadmap for institutions to implement the *NIH Single IRB Review policy*
SOA: Schedule of Activities
SOP: Standard Operating Procedure
UAMS: University of Arkansas for Medical Sciences
UC: Urgent Care
UP: Unanticipated problem
UPIRTSO: Unanticipated Problem(s) Involving Risk(s) to Subjects of Others
US: United States
